# Towards a better tomorrow: addressing intersectional gender power relations to eradicate inequities in maternal health

**DOI:** 10.1016/j.eclinm.2023.102180

**Published:** 2023-12-06

**Authors:** Meghan A. Bohren, Aditi Iyer, Aluisio J.D. Barros, Caitlin R. Williams, Alya Hazfiarini, Luisa Arroyave, Veronique Filippi, Catherine Chamberlain, Tamar Kabakian-Khasholian, Kaveri Mayra, Roopan Gill, Joshua P. Vogel, Doris Chou, Asha S. George, Olufemi T. Oladapo

**Affiliations:** a Gender and Women's Health Unit, Nossal Institute for Global Health, School of Population and Global Health, University of Melbourne, Carlton, Victoria, Australia; b Ramalingaswami Centre on Equity & Social Determinants of Health, Public Health Foundation of India, Bangalore, India; c International Center for Equity in Health, Universidade Federal de Pelotas, Pelotas, RS, Brazil; d Department of Maternal and Child Health, Gillings School of Global Public Health, University of North Carolina at Chapel Hill, Chapel Hill, NC, USA; e Department of Mother and Child Health, Institute for Clinical Effectiveness and Health Policy (IECS-Argentina), Buenos Aires, Argentina; f London School of Hygiene and Tropical Medicine, Keppel Street, London, United Kingdom; g Indigenous Health Equity Unit, School of Population and Global Health, University of Melbourne, Carlton, Victoria, Australia; h Department of Health Promotion and Community Health, Faculty of Health Sciences, American University of Beirut, Beirut, Lebanon; i Birth Place Lab, Department of Family Practice, Faculty of Medicine, University of British Columbia, Vancouver, Canada; j Department of Obstetrics & Gynecology, University of Toronto, Toronto, Ontario, Canada; k Vitala Global Foundation, Vancouver, British Columbia, Canada; l Maternal, Child and Adolescent Health Program, Burnet Institute, Melbourne, Australia; m UNDP/UNFPA/UNICEF/WHO/World Bank Special Programme of Research, Development and Research Training in Human Reproduction (HRP), Department of Sexual and Reproductive Health and Research, World Health Organization, Geneva, Switzerland; n School of Public Health, University of the Western Cape, Robert Sobukwe Road, Bellville, Western Cape, South Africa; o South African Medical Research Council, South Africa

**Keywords:** Health equity and justice, Intersectionality, Maternal health, Quality of care

## Abstract

An equity lens to maternal health has typically focused on assessing the differences in coverage and use of healthcare services and critical interventions. While this approach is important, we argue that healthcare experiences, dignity, rights, justice, and well-being are fundamental components of high quality and person-centred maternal healthcare that must also be considered. Looking at differences across one dimension alone does not reflect how fundamental drivers of maternal health inequities—including racism, ethnic or caste-based discrimination, and gendered power relations—operate. In this paper, we describe how using an intersectionality approach to maternal health can illuminate how power and privilege (and conversely oppression and exclusion) intersect and drive inequities. We present an intersectionality-informed analysis on antenatal care quality to illustrate the advantages of this approach, and what is lost in its absence. We reviewed and mapped equity-informed interventions in maternal health to existing literature to identify opportunities for improvement and areas for innovation. The gaps and opportunities identified were then synthesised to propose recommendations on how to apply an intersectionality lens to maternal health research, programmes, and policies.

**Panel: key messages**

• Maternal health inequities are some of the most pervasive in global health. While significant progress has been made in some contexts, in many others it has flatlined, and in some cases reversed due to backlash against human rights. The maternal health target of Sustainable Development Goal 3 will not be achieved if we do not adopt an intersectional approach.
• An intersectional approach illuminates how multiple forms of power and privilege (and conversely oppression and exclusion) drive maternal health inequities, the structural causes driving those inequities, and open avenues to address and unleash agency, community, and policy actions.
• We challenge the maternal and global health communities to operationalise intersectionality to imagine, invent, and co-create new approaches that move the world closer to a better, more equitable, and just future.

## Introduction

Improving access to and use of quality maternity care services as a means to reduce maternal morbidity and mortality are critical goals for maternal health. However, inequalities in access, use, and health outcomes remain persistent and profound. Exploring and responding to inequalities in coverage and use of services are important, but do not reflect the broader human rights and factors that influence women and birthing peoples’ ability to obtain dignified, quality maternity care. A more comprehensive and holistic approach to maternal health ensures a balanced consideration of pregnancy, birth, and postpartum experiences and health and well-being outcomes, as well as the quality of care received, with all being shaped by intersectional gender power relations.

Traditionally, health inequalities were studied in relation to selected social and economic parameters that were relatively easy to measure, such as wealth status, area of residence (comparing urban and rural dwellers), maternal education, and across country regions. The Sustainable Development Goals (target 17.18) call for more and better quality data that will allow exploration of other dimensions of inequality, including ethnicity, migration status, disability status, geographic location, sexual orientation and gender identity, among others.^1^ Research on health inequalities (differences in access or outcomes between population groups) compliments but is distinct from considerations of health inequities (health inequalities produced by social power relations that are unfair or unjust and should be changed) which have begun to push the field towards recognition that multiple sources of oppression operate simultaneously. These types of analyses can facilitate understanding of underlying unjust power relations, and require a more sophisticated analytical approach.

In this Series Paper, we describe how intersectional gendered power relations drive maternal health inequities and how they must be addressed. We present new inequalities analyses using an intersectional lens to illustrate the strengths and opportunities of this approach, and what is lost in its absence. We reviewed and mapped equity-informed interventions in maternal health from existing literature to identify opportunities for improvement and areas for innovation. Finally, we make recommendations to communities of research, policy, and clinical practice on how to re-imagine and co-create new intersectionality-informed approaches to maternal health, which have the potential to move us closer to health equity for all. In this paper, we use “women and birthing people” throughout as inclusive terms to reflect all populations with the reproductive capacity for pregnancy and birth (e.g., including cisgender women, and people who are transgender, non-binary, gender-fluid, intersex, and gender non-conforming). Where we use “women” only, it is to reflect the term used in existing data sources, as these data sources typically come from maternal health studies conducted with cisgender women.

## Intersectional gendered power relations that drive maternal health inequities

Intersectionality, an approach rooted in Black feminist theory and praxis,^2^^,^^3^ is well-suited to explore how different types of power and oppression operate and contribute to inequities in maternal health, well-being, and rights, while also highlighting opportunities for change. Intersectionality argues that power and privilege (and conversely oppression and exclusion) arise from multiple distinct sources that are interconnected and co-constitutive. For example, women living in poverty are doubly oppressed by both gender and class. Their experiences differ from both women who are wealthy and men living in poverty, who simultaneously experience both privilege and oppression.^4^ Gender restricts the ability of women living in poverty to access resources that could shield them from adverse effects of poverty (e.g., stable employment), even as poverty undermines their ability to protect themselves from gender oppression (e.g., leaving a violent relationship). Importantly, the effects of multiple intersecting oppressions and/or privileges are neither summative nor multiplicative, because the intersections are complex–especially when advantages interact with disadvantages. Consequently, effect sizes cannot be quantified by knowing only the effects of each individual dimension.

Intersecting power relations shape people—their social identities, experiences—and the social norms, values, and moral codes through which their lives are governed within households, communities, and schools. They also play out through the rules and policies defined and executed by legal, governance, education, and health systems, among others. Finally, intersectionality posits that systems of power, with the ideologies that created and support them, are attuned to historical, political, economic, and cultural contexts.^5^ Systems of power likewise vary over time and across socio-cultural and geo-political borders.^5^

Intersectionality foregrounds inequities and injustices that mark maternal health, and contrasts with how unidimensional inequalities flag—but do not adequately represent—inequities and injustices. Pregnancy and childbirth are explicitly tied to sexuality, reproductive health, and human rights, which are governed by heteronormative gendered power relations. These gendered power relations rationalise and perpetuate unequal access to and control of resources. They can lead to skewed divisions of labour (e.g., child-rearing), and familial decision-making leaving women and birthing people disempowered. Gendered power relations are sustained by discriminatory social norms or even the threat of (or actual) gender-based violence, all of which militate against maternal safety and rights.^6^^,^^7^

Using an intersectionality approach allows for explicit exploration of how gendered power relations interact with other sources of inequality to shape or impair agency, cultural expectations, and access to resources, support, and care during the perinatal period. In some gender inequitable societies, girls may be pushed into early marriage and childbearing, especially among families living in poverty in rural areas.^8^^,^^9^ Similarly, unmarried and adolescent mothers, who may be more likely to live in poverty, describe feeling socially stigmatised and mistreated by healthcare institutions and providers.^10^^,^^11^ Women and birthing people may have limited agency and bodily autonomy throughout pregnancy and childbirth; for example, limited autonomy over the decision to seek healthcare or financial resources to do so,^12^^,^^13^ or fear that gendered assumptions about pregnant bodies may result in lack of gender-affirming healthcare.^14^ They can be mistreated during childbirth, particularly where there are organisational challenges to providing care^15^ or lack of person-centred maternity care.^16^, ^17^, ^18^, ^19^, ^20^ Many maternity services use gender-biased curricula and obstetric practices, or devalue midwives and nurse-midwives, which are historically female-dominated professions.^21^, ^22^, ^23^ Women and birthing people as both providers and users of maternity care services bear the brunt of this gendered discrimination. Panel 1 provides illustrative prompts that can be used to explore and address gendered power relations in maternal healthcare services, based on adaptations of gender analysis-informed research in Myanmar and Uganda.^6^^,^^7^^,^^24^

*Panel 1* **Exploring and addressing gendered power relations in maternal healthcare services.**

This panel presents examples of illustrative prompts to explore how gendered power relations can cause inequities and harm within maternal healthcare services. Using these prompts can help policy-makers and service providers think through how they can address inequities and improve service design and delivery. They are based on materials adapted from gender analyses and research in Myanmar and Uganda.^6^^,^^7^^,^^24^

**Access to resources**

• Do health facilities provide services with appropriate physical conditions?
• Are health workers trained and experienced to work with specific populations (e.g., people who are transgender or non-binary, migrants, or refugees)?
• Are services available and accessible to all people who need them (e.g., regardless of marital status, age, language, disability, gender identity)?
• Are marginalised populations able to access information and care (e.g., minoritised ethnic groups, transgender people, non-binary people, gender non-conforming people, people living in poverty, migrants without legal identity)?
• Are there services to identify and respond to gender-based violence in a sensitive and effective manner?
• Are there services to support pregnancy loss and abortion care?
• Are services financially accessible for all?
• How are women and birthing people with low health literacy supported to make informed decisions about their health and care?
• How do staff react when women or birthing people who are unbooked for birth arrive, or arrive without antenatal care cards?
• Are there policies that allow a labour companion of the woman's or birthing person's choice, and practical actions to ensure that all who want a labour companion are able to have one?
• How is privacy ensured during examinations, labour, and childbirth?
• Are staff paid with adequate wages received on a timely and consistent basis?
• What can be done to ensure staff are supported by colleagues, supervisors, and the work environment to promote health working environments and avoid burnout?
• What other barriers affect access to maternal healthcare services?

**Division of labour and everyday practices**

• Are maternal health services organised in a way that considers women's and birthing people's agricultural, economic, and care-taking needs?
• Are women and birthing people asked or expected to clean up after themselves after birth?
• Are women and birthing people chastised by staff for poor hygiene?
• Are staff without appropriate qualifications asked or required to provide care (e.g., cleaners)?
• How well do staff of different cadres collaborate, for example, midwives and obstetricians? Do current structures empower or disempower cross-cadre collaboration and respect?
• How well do health facility administrators and managers negotiate everyday management of the health facility, including procurement, governance, and staffing?

**Social norms**

• What are women's, birthing people's, and community's preferences about place of birth (birthing in the community, at home, or at a health facility), mode of birth (vaginal, caesarean section), desire for pain management interventions, etc?
• How can staff and health facilities help women and birthing people to maintain safe cultural practices?
• Do services encourage the participation of men in maternity and paediatric healthcare? If yes, how and on what terms?
• What are the perspectives of women, birthing people, communities, and staff related to mistreatment during childbirth and gendered violence?
• To what extent are certain health conditions normalised (e.g., HIV, sexually transmitted infections, adolescent pregnancy)?
• Do providers normalise non-evidence based procedures (e.g., promoting caesarean sections in situations where they are neither medically indicated nor desired by the woman or birthing person, routine perineal shaving?)

**Rules and decision-making**

• Who decides whether and how much of household resources to allocate to maternal healthcare services?
• Do women or birthing people need to get permission from a male partner or family member to visit a health facility?
• Do policies exist to promote gender-responsive health systems?
• Are maternity care services covered by insurance, free-of-charge, or covered by non-governmental organisations?
• Are informal payments or bribes expected at the point-of-care?
• Are women and birthing people expected to comply with all decisions made by healthcare providers, even if they disagree or do not understand?
• Are there formal or informal rules that govern who is allowed to visit the labour, delivery, and postnatal wards, and at what time of day they are allowed that might inhibit labour companionship?
• Are women and birthing people allowed to mobilise throughout labour and do they have easy access to oral fluids and food?
• Is there adequate and easy access on the labour and delivery wards to pharmacological and non-pharmacological methods of pain relief?
• Are women and birthing people allowed to birth in a position of their choice, or only in the lithotomy position?
• Do women and birthing people who have a caesarean birth receive sufficient information on the risks and benefits of caesarean versus vaginal birth? Were informed consent and debriefing processes adequate?
• What accountability mechanisms are in place? Who do they feed back to?

Other deep-rooted sources of discrimination further intensify gendered discrimination, including racism, casteism, ageism, ableism, and transphobia.^25^ Intersecting effects of “gender and racism” or “gender and casteism” can be so strong that economic class may afford little—if any—alleviation. There are many instances of racism deeply ingrained into health and social policies that govern maternal health and healthcare. For example, gendered racism is revealed and persists in stereotypes that stigmatise Black motherhood.^26^ Gendered racism can manifest with health workers labelling Black women as “difficult patients”,^27^ or maternity care practices that leave Black women feeling unsafe, unheard, or dismissed,^28^ all contributing to persistent racial inequities in maternal health outcomes and experiences.^29^ This gendered racism has historical roots—enslaved Black women were surgically experimented on without their consent or pain management by the “father of modern surgical gynaecology” James Marion Sims in the 1800s.^30^ Gendered exploitation of enslaved Black women who were forced to breastfeed the children of white women (wet-nursing) was also commonplace.^31^ In Australia, government policies forcibly removed Aboriginal and Torres Strait Islander babies and children from their families, placing them with non-Indigenous families (Stolen Generations).^32^^,^^33^ These experiences have led to compounding cycles of intergenerational trauma, including broken cultural, spiritual, and family ties, distrust in health systems, and inequitable health and well-being outcomes.

Intersectionality as an analytic approach encourages moving beyond overly-simplistic conceptualisations and measurements of inequality. Intersectionality analysis can be used to explore how social identities and structures intersect and contribute to power/privilege and oppression/exclusion.^34^ Within maternal health, an intersectional approach encourages consideration of women and birthing people as agents of the powers that govern their lives—rather than a sum of their social identities (e.g., sexual orientation, race, caste).^35^^,^^36^ Intersectionality can therefore contribute to deeper, more nuanced understanding of how and why women and birthing people can simultaneously be privileged yet disempowered.

## Applying an intersectional lens to monitoring a maternal health indicator

To illustrate the utility of intersectionality analysis in maternal health, we used a novel indicator of antenatal care (ANC) quality: the ANCq8+ indicator.^37^ The overall ANCq indicator includes information on contact with the health service (number of ANC visits, timing of first ANC visit) and content of care (provider qualification, collection of blood and urine samples, blood pressure measurement and tetanus shot in current pregnancy). ANCq scores range from zero (no ANC) to ten (best care based on the items), and we used the proportion of women with a score of eight or more (ANCq8+) as a proxy of good quality ANC.

We selected ANCq8+ as ANC is the entry point to maternal healthcare services, has important implications for the woman or birthing person and baby, and is an indicator where reliable data can usually be obtained. We focused on a quality indicator, rather than a health outcome or access indicator, to reflect contemporary shifts in maternal health to improve quality of care.^38^, ^39^, ^40^, ^41^ Using national health surveys conducted since 2015 (Demographic and Health Survey [DHS] and Multiple Indicator Cluster Surveys [MICS]), we explored the quality of ANC received by women in Latin America and the Caribbean, across different ethnicities and socioeconomic deprivation status (SDS, a multi-dimensional approach to measuring poverty^42^). The main aim was to use an intersectionality-informed approach^43^^,^^44^ to assess how population groups with a double burden of disadvantage compared to other more advantaged groups. Supplementary Methods 1 contains further details on the methods and descriptive results.

We restricted our analyses to eight Latin America and Caribbean countries (Belize, Cuba, Guyana, Honduras, Mexico, Paraguay, Peru, and Suriname) where we could standardise ethnicity to: Indigenous, Afro-descendant, and others (not identified by any of the previous groups). We estimated the SDS based on eight items from two domains: education (school-aged children in school, at least six years of education for adults) and living standards (non-use of solid fuel for cooking, sanitation facilities, safe drinking water source, electricity, assets, and adequate housing materials). Each item not available added a point to the score, the final score composed of the two domain scores equally weighted.^42^ We divided the sample into approximate tertiles of “more deprived” (the top deprivation tertile), and “less deprived”.

**Fig. 1:**
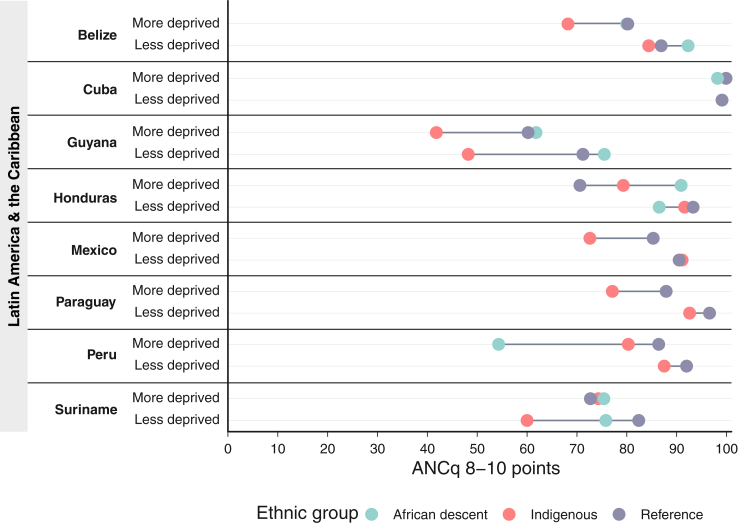
**Antenatal care quality by ethnicity and socioeconomic deprivation status in Latin America and the Caribbean**. This equiplot demonstrates that, in general, women who are more socioeconomically deprived tend to receive lower quality antenatal care (ANC) compared to less deprived women. When socioeconomic deprivation is combined with ethnic disadvantage, it further contributes to a decrease in the quality of ANC. The equiplot underscores the significance of employing intersectionality-informed analytic approaches, as these approaches recognise that the overlapping oppressions of ethnicity and socioeconomic deprivation cannot be simply addressed by combining multiple one-dimensional measures of deprivation. Reference population are women who were not identified in the Indigenous or Afro-descendant groups.

Fig. 1 shows that, generally, the more deprived groups had a lower proportion of women with quality ANC than the less deprived. This demonstrates the critical role of socioeconomic class, which modifies ethnicity-based inequities to varying degrees in all countries except Cuba. Socioeconomic deprivation combined with ethnic disadvantage served to push these women below national averages and their less deprived counterparts in all countries except Suriname (among Indigenous women) and Honduras (among Afro-descendent women). In the context of ANCq8+, being an Indigenous woman and more deprived represents a burden that is disproportional to just being Indigenous or deprived alone. In nearly all cases, the disadvantage of Indigenous women regarding ANCq8+ are larger among those who are more deprived. Cuba represents a remarkable example of equity and quality ANC, with ANCq8+ close to 100% for all women. These findings align with intersectionality theory's assertion that intersecting oppressions are not equivalent to combining multiple unidimensional measures of deprivation. Rather, these intersecting oppressions create situations of “double jeopardy,” where women and pregnant people must simultaneously navigate multiple sources of oppression.

## Equity-informed maternal health interventions

Recognising that we could measure the effects of intersecting oppressions and privileges in our data, we next sought to deepen understanding of how intersectionality- and equity-informed approaches have been used in existing maternal health interventions. We conducted a scoping review to systematically identify and describe interventions that use intersectionality- or equity-informed approaches to address social power relations or inequities in maternal health (protocol registration: osf.io/9fyhc, Supplementary Methods 2 describes full methodology). We defined “equity-informed interventions” as those that included mechanisms of action that directly or explicitly aimed to reduce inequalities or promote equality in maternal health or maternity services (e.g., voucher programmes for free antenatal care targeting those with low income, community mobilisation for marginalised communities to improve healthcare access and use). We defined “intersectionality-informed interventions” as those that explicitly stated the application of intersectionality in designing or implementing an intervention or programme (e.g., whether the intervention was based on identifying a problem using an intersectional perspective such as being representative of the experiences of diverse populations, or designed to lead to a change in power relations).^45^^,^^46^ Briefly, we included studies that 1) were an intervention to promote equality or equity, reduce inequality or inequity, or used an intersectionality-informed approach in maternal health or healthcare settings, 2) were randomised or non-randomised trials, pre-post studies, interrupted time series, realist evaluations, or other study designs comparing interventions with usual care, and 3) included quantitative evaluation. We excluded studies that conducted an equity/inequity analysis of an intervention, but where the intervention was not explicitly designed to reduce inequity or promote equity (e.g., secondary analyses of the impact of interventions on different population groups), as we considered embedding equity in intervention design critical to ensure no one is left behind. We searched MEDLINE and CINAHL using structured search terms, from inception to May 23, 2022.

**Fig. 2:**
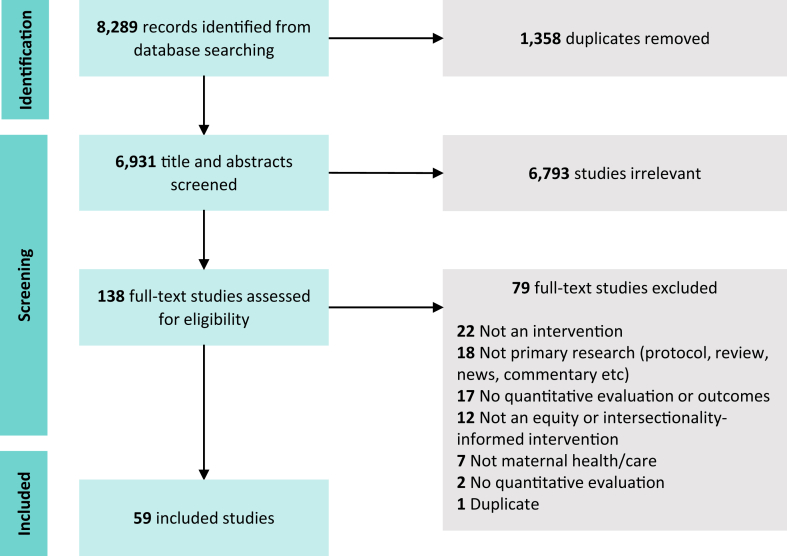
**PRISMA flowchart depicting scoping review search and selection process**.

We identified 8289 citations from the database searches and included 59 studies that were published between 2008 and 2022 (Fig. 2: PRISMA flowchart). Supplementary Table S1 reports a summary of the included studies; Supplementary Table S2 reports characteristics at the study-level. In summary, the 59 included studies were conducted in 31 countries across all regions, predominantly in Southeast Asia and Africa (43 studies, 74.6%). Most studies were conducted in low- (19 studies, 30.6%) or lower-middle income countries (33 studies, 53.2%).

### Characteristics of equity-informed interventions

Supplementary Table S3 reports a summary of the intervention characteristics in the included studies; Supplementary Table S4 reports intervention characteristics at the study level. Most of the 59 included studies were randomised trials or quasi-experimental studies (44.1%), and varied in terms of the maternal health topic, with 38 studies (64.4%) focusing on access to and use of maternal healthcare services (ANC, childbirth care, and postnatal care), five studies (8.5%) about maternal health quality of care (respectful care, integrated maternal and newborn care, cultural safety), five studies (8.5%) about health systems and governance (digital health interventions for continuum of care, community mobilisation, maternity protection laws, maternity leave), four studies (6.8%) about maternal and child nutrition, four studies (6.8%) about postnatal and neonatal health (preterm birth, low birthweight, breastfeeding, neonatal mortality), and three studies (5.1%) about modern contraceptive uptake.

All studies had explicit aims to improve equity through the programme and/or intervention design, most commonly via health behaviour change interventions (e.g., community health promotion, strengthening community-health facility connections), introduction of free or subsidised maternity care services, and conditional or unconditional cash transfers. Approximately half of the included studies specifically targeted underserved or marginalised groups, most commonly women experiencing poverty, Indigenous women, women living in rural areas, women from ethnic minority backgrounds, or women experiencing food insecurity. Almost all studies used equity-informed outcome evaluation, typically wealth status, education, age, urban or rural place of residence, or caste.

### Substantial room for PROGRESS in intervention design and evaluation

While we aimed to identify both equity- and intersectionality-informed interventions in maternal health in the scoping review, only equity-informed interventions were found. In order to explore the types of equity considered in the type of interventions, target populations, and outcomes of the included studies, we mapped the 59 included studies to the PROGRESS-Plus social factors (Table 1). PROGRESS-Plus is an acronym used to illustrate a sample of social stratifying factors that may account for inequitable variations in health and well-being outcomes, and is increasingly used in equity analyses in both interventions and systematic reviews.^47^^,^^48^ Specifically, PROGRESS-Plus stands for **p**lace of residence (e.g., urban/rural, country, region), **r**ace/ethnicity/culture/language, **o**ccupation, **g**ender or sex, **r**eligion, **e**ducation, **s**ocioeconomic status, **s**ocial capital, personal characteristics or identities associated with discrimination (e.g., age, disability), features of relationships (e.g., parents who are divorced), time-dependent relationships (e.g., discharge from hospital following birth, time periods where people may temporarily be at a disadvantage). While the PROGRESS-Plus social factors are not exhaustive, they illustrate the multi-dimensionality of social identities and factors that may be addressed explicitly, measurably, and rigorously in interventions to improve health equity, and can help operationalise intersectionality in practice.^47^

**Table 1:** Assessing the extent to which PROGRESS-Plus social factors are considered in global maternal health interventions.

	Intervention design **n = 59 studies**^a^		Equity-informed target population **n = 31 studies**^b^		Equity-informed outcome evaluation **n = 51 studies**^c^	
	n (%)	PROGRESS-Plus factor	n (%)	PROGRESS-Plus factor	n (%)	PROGRESS-Plus factor
Place of residence	4 (6.8%)	Targets access in rural and remote areas	4 (12.9%)	Resident in rural area	13 (25.5%)	Urban/rural, geographic access to facility
Race, ethnicity, culture, language	3 (5.1%)	Targets racial disparities and cultural safety	5 (16.1%)	Indigenous identity, ethnic minority	12 (23.5%)	Caste, race/ethnicity, Indigenous identity
Occupation	2 (3.4%)	Paid parental leave	1 (3.2%)	Women who work	5 (9.8%)	Employment status
Gender/sex	2 (3.4%)	Promote gender equity	1 (3.2%)	Women and men	4 (7.8%)	Gender
Religion	0 (0.0%)	–	0 (0.0%)	–	2 (3.9%)	Religion
Education	7 (11.9%)	Health education	0 (0.0%)	–	16 (31.4%)	Education, literacy
Socioeconomic status	28 (47.5%)	Vouchers, fee-subsidies, free services, user fee reductions, cash transfers	19 (61.3%)	Poverty, food insecurity	41 (80.4%)	Wealth status
Social capital	14 (23.7%)	Safe motherhood action groups, community mobilisation, community health and health extension workers	0 (0.0%)	–	0 (0.0%)	–
Refugee/migration status	1 (1.7%)	Professional development, group antenatal care	1 (3.2%)	Women of refugee backgrounds	1 (2.0%)	Refugee status
Age	0 (0.0%)	–	0 (0.0%)	–	11 (21.6%)	Age
Justice-involved individuals	1 (1.7%)	Justice reform	0 (0.0%)	–	0 (0.0%)	–

This table maps the studies included in the scoping review to PROGRESS-Plus social factors (rows), based on whether the PROGRESS-Plus social factor was addressed in the intervention design, target population, or outcome evaluation. The table shows that socioeconomic status is the most commonly addressed social factor across intervention design, target population, and outcome evaluation, while more work is needed to address other social inequities and their intersections.

a All 59 included studies.

b 31/59 included studies that specifically targeted a historically underserved or marginalised population.

c 51/59 included studies that conducted equity-informed outcome evaluation (e.g., disaggregating data by equity identifiers or calculating concentration indices).

We found that socioeconomic status was by far the most common factor accounted for in almost half of intervention designs (e.g., vouchers, fee subsidies, cash transfers), over half of target population (people experiencing poverty or food insecurity), and over 80% of outcome evaluations (wealth status). Few interventions specifically aimed to improve racial or ethnic inequalities or targeted people from ethnic minorities or Indigenous groups. While most equity-informed outcome evaluations presented analyses by wealth status, only approximately one-quarter of studies reported outcomes based on place of residence, age, or race, ethnicity, culture, or language, and less than 10% of studies reported outcomes based on employment status, gender, religion, or migration or refugee status. These analyses suggest that most equity-informed interventions in maternal health focus on addressing and measuring financial equity, while more work is needed to address other forms of social inequities and their intersections. We discuss the implications of these analyses in the following sections.

### Lack of intersectionality-informed interventions

We did not identify any intersectionality-informed strategies or interventions in global maternal health that were measured with quantitative approaches, either from the perspective of intervention design or outcome evaluation. This represents a major gap and recommendation for future research and intervention design. None of the included studies used intersectionality-informed analyses, which might have shed further light on the people's multiple, dynamic, and contextualised social identities, how they are located within broader structural conditions that must be addressed, and how agency at individual- and group-levels can be supported to address these power relations. Intersectionality-informed intervention design has the potential to encourage researchers, program managers, and policy-makers to critically examine the relationships and interactions between social identities (e.g., PROGRESS-Plus factors), structural systems, and power relations that represent fundamental causes of health equity and inequity. In the context of maternal health, intersectionality-informed intervention design may encourage reflection on the diversity of women and birthing people in terms of pathways to pregnancy, unique healthcare needs, and life circumstances. Understanding these nuances is critical to achieve truly person-centred maternity care, and encourage us to move away from thinking of people solely as so-called vulnerable populations or stigmatised groups who need additional care, to instead prioritise collective ways to reassess strategies and support agency across the health system to transform such realities.

### Over- and under-representation of dimensions of equity

Most interventions identified in the scoping review addressed financial (poverty) and place (rural, far distance from health facilities) inequities in design, population, and outcome evaluation. While addressing financial and place inequities are critical to leaving no one behind, more work is needed to understand the complexities of these inequities and how other social identities such as migration or refugee status, Indigenous identity, disability, sexual orientation, and gender identity may reinforce or alleviate power imbalances. For example, rural poverty is inherently different from urban poverty, and addressing barriers to healthcare access and use, and fostering positive experiences of maternal healthcare require different approaches. Inequality analysis alone is therefore likely to underestimate or simplify the challenges faced, and thus solutions proposed.

### Equity beyond access to maternal healthcare services

Two-thirds of studies focused on improving access to and use of maternal healthcare services, and only five studies focused on improving quality of care, including experiences of and satisfaction with care. Given the strong existing evidence of racism and discrimination in maternal healthcare and influence of poor experiences of care on health, well-being, and future health-seeking behaviours,^49^ more interventional work is urgently needed to address these important, neglected components of maternal health. Measuring equity and experiences of care (including discrimination) is particularly important in evaluating maternal health interventions that target specific marginalised groups. Although well-intended, if these person-centred outcomes are not measured then it will not be possible to ascertain whether improved access to care for marginalised groups simultaneously resulted in further discrimination within healthcare settings.

## Recommendations for practice, research, and policy

Much has been accomplished by increasing access to and coverage of maternal healthcare services, with substantive reductions in maternal and newborn mortality. However, a substantial unfinished agenda remains to ensure maternal well-being, rights, and justice for all.^50^ While maternal health inequities are well-recognised, understanding fundamental causes of and potential solutions to these inequities remain less understood and resourced. Using an intersectionality lens can help to improve maternal healthcare services, enable choices that cater for diverse needs, and yield critical insights and new strategies to ensure universal access to high quality, respectful, and person-centred maternity care. To meet the ambitious objectives articulated in the Sustainable Development Goals and codified within the right to health, a broader vision that embraces the central role of power relations^50^ in maternal health is urgently needed. In the following sections, we propose a way forward to apply an intersectionality lens to maternal health practice, research, and policies.

### Embedding intersectionality into clinical practice

Establishing intersectionality as a grounding principle of clinical practice provides a way forward to improve maternal health and healthcare across the full spectrum of conditions and services (including pregnancy and birth experiences, ANC, perinatal care, postnatal care, mental healthcare, self-care, abortion, stillbirth, preterm birth, and miscarriage). Yet this will require medical institutions themselves to recognise the power structures within medicine and healthcare that reinforce inequities. This requires reflection and action to reckon with power and privilege within medicine itself, including the structures that favour racialised white, cisgender and heterosexual men.^51^

Moving beyond a biomedical approach to maternal health and healthcare, using an intersectionality lens in training programmes can provide opportunities for people to evaluate the gendered power relations that create inequities and, if not addressed, can further be amplified within healthcare settings.^51^ Similarly, embracing multi-disciplinarity, trauma-informed, and social justice approaches to maternal health can provide a way forward to improve maternal health and healthcare. Examining the role of unconscious bias, including how to recognise and challenge it, should be embedded within clinical training curricula.

Person-centred care charters (e.g., the Respectful Maternity Care Charter^52^) publicly declare the health facility values and establish baseline expectations for care. Adopting these practices can help align health facilities and health systems with cross-cutting values of quality, equity, and human rights. Moreover, communities need mechanisms to hold health workers and facilities accountable when mistreatment and discrimination do occur. Community scorecards^53^^,^^54^ and health facility-rating apps^55^ are mechanisms for generating demand for accountability, building trust, and improving person-centred dimensions of quality care. Lastly, incorporating measures of peoples’ experiences of care into quality improvement efforts^41^^,^^56^ can help normalise considerations around respectful care as key aspects of quality care. These approaches to re-envisioning clinical practice can be complemented by revitalised research into health inequities and social justice.

### Catalysing research on intersectionality and maternal health justice

Intersectionality offers a more nuanced and context-responsive lens to conceptualise power/privilege and oppression/exclusion, and is responsive to limitations in existing inequalities research. Despite nominal recognition of intersectionality within maternal health, much maternal health research relies on unidimensional measures that belie the complexity of human lives. Moreover, this research is largely deficit-based, focusing on describing inequalities or vulnerabilities, while ignoring the strengths, agency, and potential levers to empower women, birthing people, and communities to mobilise for a better future. Intersectionality analysis therefore also offers an opportunity to illuminate the negatively compounding factors and enable consideration of how these can be transformed into positively reinforcing factors to improve health equity. This is particularly critical during the perinatal period, which is a unique life-course opportunity for improving health equity for women, birthing people, babies, and families.

A substantial body of evidence describes and measures poor experiences of maternity care including mistreatment and discrimination^16^^,^^19^^,^^56^^,^^57^; however, our scoping review shows that equity-informed interventions in maternal health remain focused on coverage (access and use) rather than quality of care. These interventions typically conceive of inequity as a unidimensional concept, conflating poverty with inequity. In contrast, an intersectional approach highlights the important contribution of poverty to inequity, without neglecting other important personal and social conditions (e.g., rurality, race/ethnicity, LGBTQIA + identity).

Despite these challenges, there are other ways that research has the potential to revolutionise maternal health. Feminist, critical, and decolonial ontologies reorient inquiry around systems of resistance and oppression, provide new mental models for theorising effects of just or oppressive systems,^50^^,^^51^^,^^58^, ^59^, ^60^, ^61^ and spur the development of more sophisticated approaches to assessing the impact of said systems on maternal health. Qualitative, community-based participatory, arts-based, human-centred design, and action research methods centre the embodied experiences of research participants and engage them as co-designers and co-interpreters of data. These emancipatory research methods can both democratise knowledge creation and strengthen opportunities for community-led action.

Narratives of pregnancy, labour, and birth are layered, complex, sensitive, and deeply embedded in the narrator's positionality,^12^^,^^18^^,^^19^^,^^21^ which influences their experiences, expectations, and perspectives of respect or mistreatment during maternity care. Qualitative arts-based methods such as body mapping^62^^,^^63^ and I-poetry^64^ provide unique tools to engage with participants with ample flexibility for the participants to decide the course and content of the research. These approaches can be coupled with analysis methods including feminist relational discourse analysis^62^ and voice-centred relational analysis,^64^ which aim to shift power from the researcher's interpretation to the participant voices.

Translating these complexities into operational indicators—and on a global scale—represents a substantial research gap. We demonstrate the value of intersectionality analysis using an ANC quality indicator to improve documentation of inequalities at varying intersectional positions, and explore potential individual- and group-level drivers of observed inequalities.^43^ The levels of advantage or disadvantage of each group varies widely across countries, demonstrating the importance of context-specific analyses. As such, we advocate for greater inclusion of intersectionality analyses in maternal health in order to move beyond unidimensional equity categories to make visible these intersecting identities and social positions to inform the development of interventions and policies. Far from an obstacle, this is a rich opportunity for methodological innovation.

Our focus here on research methodology should not obfuscate the fundamental question: who should maternal health research serve? If the purpose is indeed to ensure the health and well-being of all women, birthing people, babies, and families then our research must answer the pressing questions facing them, their families and communities, and the policy-makers answerable to them. In other words: research must be relevant, applicable, and actionable, and researchers must see translation into policy and practice as their central mandate.

### Re-imagining the global maternal health community

Policy plays a critical role in creating and upholding power relations that drive inequities, and can thus be a powerful tool to rectify inequities. Policy operates at institutional, subnational, national, regional, and global levels, and all are useful levers to engender change. Facility- or health system-level policies that regulate staffing allocations, clinical hierarchies, and available equipment and infrastructure all influence health workers' abilities to provide person-centred maternity care. For example, qualitative research in Argentina found that the Ministry of Health's policy to create private labour rooms improved health workers' abilities to provide respectful maternity care.^65^ Similarly, increasing staff pay, improving staffing ratios, and addressing health worker burnout can create more enabling environments for quality care. Sub-national and national policies also have the potential for transformative change. For example, repealing policies criminalising LGBTQIA+ people or barring them from “women-serving” spaces (toilet facilities, maternity wards) can reduce access barriers to maternal healthcare services.

Professional associations, accrediting bodies, and licensing organisations can support efforts to embed intersectionality into clinical practice. For example, they can support updates to pre-service curricula or provide incentives for in-service education through existing in-service or continuing education systems. These institutions can also provide targeted mentorship through affinity groups for professionals from marginalised communities, to help diversify the maternal health workforce. They can also take action to ensure that their leadership reflects the diversity of communities they serve, and meaningfully engage with the resilience and resourcefulness of these communities.

At the global level, human rights approaches for health provide one avenue for engaging with intersectionality. The United Nations Special Rapporteur on the right to health has framed her mandate through the lens of intersectionality, illustrating how legacies of colonialism and other oppressive systems produce adverse outcomes, including in maternal health.^66^ Intersectionality also allows for an understanding of overlapping State obligations for members of multiply marginalised populations. For example, States may have legal obligations to rectify inequities due to gender and disability under both the Convention on the Elimination of Discrimination against Women and the Convention on the Rights of Persons with Disabilities, underscoring the imperative to ensure there is accessible quality maternity care available for women and birthing people with disabilities.

## Conclusion

Applying an intersectionality lens to maternal health has implications for framing clinical practice, research, and policy, and has the potential to improve understanding—and therefore action—to improve health and well-being for all, especially those marginalised by systems of power. This means moving from a one-size-fits-all approach to more contextually-relevant approaches that enable community-led design and implementation to redress historical power imbalances.

Many of the maternal health inequities described throughout this Series Paper are viewed by the global and maternal health communities as entrenched and immovable. Yet these inequities are entrenched only in so far as the global health community continue to leave underlying structural drivers unaddressed. We have offered a set of possible new research, policy, and clinical practice approaches that seek to address the challenges of eradicating entrenched inequities. This list is not exhaustive and should not be viewed as a checklist for “doing intersectional equity for maternal health.” Indeed, there is no single way to assure intersectional equity will work for all people and in all settings. Rather than presenting a playbook, we hope these reflections spur the global health community, and specifically the maternal health community, to imagine, invent, and co-create new approaches that move the world closer to a better, more equitable, and just future.

## Contributors

OTO and MAB conceptualised the series paper. AJDB and LA conducted the analysis of DHS and MICS data, with inputs from AI and MAB. VF, CC, TKK, AH, and MAB conducted the scoping review. All authors contributed to data interpretation. AJDB, AI, CRW, and MAB prepared the first draft of the manuscript. AJDB and LA have verified the underlying data for the DHS and MICS analysis. MAB and AH have verified the underlying data for the scoping review analysis. All authors had full access to all data in the study, and all authors critically reviewed, revised, and approved the final version of the manuscript.

## Data sharing statement

The datasets used for the intersectionality analysis of Demographic and Health Survey (DHS) data are available from the DHS Program website (https://dhsprogram.com). The dataset generated for the scoping review is available in the manuscript and Supplementary materials.

## Declaration of interests

We declare no competing interests.
