# Supplementary material for: Towards a better tomorrow: addressing intersectional gender power relations to eradicate inequities in maternal health
Source: eClinicalMedicine. 2023 Dec 6;67:102180. doi: 10.1016/j.eclinm.2023.102180 (PMC10837533; doi:10.1016/j.eclinm.2023.102180)
Supplement: Supplementary Appendix [file mmc1.pdf]

Supplementary appendix

1. Supplementary methods 1 – DHS and MICS analysis of ANCq8+

2. Supplementary methods 2 – Scoping review methods on equity-informed maternal health interventions

3. Supplementary Table 1 – Scoping review summary of characteristics of included studies

4. Supplementary Table 2 – Scoping review characteristics of included studies at the study-level

5. Supplementary Table 3 – Scoping review summary of intervention description and outcomes

6. Supplementary Table 4 – Scoping review intervention description and outcomes at the study-level

Supplementary methods 1: DHS and MICS analysis of ANCq8+

This supplementary methods section describes the methodological approach used for the DHS and MICS analysis of the ANCq8+ indicator.

Data sources

We used data from Demographic and Health Surveys (DHS) and Multiple Indicator Cluster Surveys (MICS), which are nationally representative household surveys carried out in low- and middle-income countries (LMICs) with focus in reproductive, maternal and child health, and nutrition. DHS and MICS use standardised data collection procedures, comparable across and between surveys and countries.[1-3]

We included in the analysis all countries from the Latin America and the Caribbean (LAC) region which had a DHS or MICS survey carried out in since 2015 with information on ethnicity (or a proxy indicator such as language or skin colour) and the variables needed to calculate the ANCq – a content-qualified antenatal care (ANC) indicator. We identified eight countries in LAC with data for the analysis – Belize, Cuba, Guyana, Honduras, Mexico, Paraguay, Peru, and Suriname. The most recent survey for each country was selected when more than one survey had been carried out since 2015.

In our analyses we explored how the ANCq varied according to ethnicity and levels of multidimensional poverty using an indicator adapted for equity analyses. This indicator – the socioeconomic deprivation status (SDS) – is based on the global MPI (Multidimensional Poverty Index).

Maternal health indicator: ANCq

ANC was assessed using the ANCq, a novel survey-based ANC indicator calculated as a score, that combines aspects of contact with the health service (number of ANC visits, timing of first visit) and content of care received (provider qualification, collection of blood and urine samples, blood pressure measurement and tetanus protection). The ANCq score ranges from zero, for women with no ANC, to 10 points, for women getting the best care based on the items. The ANCq has been validated, showing significant inverse associations with neonatal mortality. Full details on the construction of the indicator and its validity are presented elsewhere.[4] For the analyses presented in this paper, the ANCq score was dichotomised into score eight or more, or less than eight, and indicated as ANCq8+. The global cut-off point was based on the global median of the ANCq of seven, so ANCq8+ is above the global median.

Stratifiers

In this paper, we performed stratified analyses to explore the intersectionality between ethnicity and indicator of multidimensional poverty (SDS) in relation to the ANCq.

Ethnic group

We focused our analyses in the LAC region where three main ethnic groups can be recognised – Indigenous people, Afro-descendants, and others not identified by any of the previous groups. The first ethnic group includes Indigenous women identified by either self-reporting being part of one of their country's Indigenous nations or by the language spoken at home. The second includes women classified as Afro-descendants, descending from the 12.5 million African people brought to the Americas as enslaved people between 1500-1867. Finally, the third group is composed by the remaining population not identified as Indigenous or Afro-descendants (this group was used as the reference in our analyses), mostly comprising European coloniser descendants and subsequent waves of migration, people with mixed-ethnicity, and more recent migrants.

These countries present very different ethnic compositions, and the population proportions in each group varied widely. Honduras and Paraguay had the highest proportions of Indigenous population (69% and 42% in our sample) and Cuba and Suriname had the highest proportions of Afro-descendants (almost 35% in each country).

Socioeconomic deprivation status (SDS)

The SDS is an indicator of multidimensional poverty adapted to equity analyses, based on the global MPI (Multidimensional Poverty Index, https://ophi.org.uk/multidimensional-poverty-index/) It is calculated using eight indicators from two MPI domains – education (two items) and living standards (six items).

Educational indicators were related to school attendance of school aged children and at least six years of education for household members. Living standards considered the use of solid fuel for cooking, access to sanitation facilities, safety of the drinking water source, electricity, assets, and adequate housing materials. The items are dichotomous indicators of deprivation (e.g. no household member has completed at least six years of education) and a score is derived from them combining all the indicators where each dimension accounted for 50% of the weight of the score and each indicator had equal weights within each dimension. Full details on the construction of the indicator are presented elsewhere.[5] Based on these scores, we divided the samples into approximate tertiles. The top deprivation tertiles was classified as "more deprived" and the two first tertiles as "less deprived". The proportions in each group and country are not always close to 1/3 and 2/3 due to ties in the classification of households.

Analysis

The proportions of women with 8 or more ANCq points for more and less deprived groups in each ethnic group for each country were calculated, taking into account the survey design including sample weights, clusters and strata. The analyses were performed using Stata (StataCorp. 2019. Stata Statistical Software: Release 17. College Station, TX: StataCorp LLC).

Ethics

The analysis was based on anonymised publicly data which is available after a registration process. Ethical clearance was done by each of the institutions responsible for carrying out the original surveys. Patients or the public were not involved in the design, conduct, reporting, or dissemination plans of our research.

Intersectionality analysis of antenatal care quality in Latin America

Country	Year	Source	Ethnic group	ANCq 8+ proportion		
				Less deprived (Tertile 1-2 SDS)	More deprived (Tertile 3 SDS)	All
Belize	2015	MICS	Indigenous	84.4	68.2	73.7
			African descent	92.3	80.0	91.3
			Reference	86.9	80.2	85.4
Cuba	2019	MICS	African descent	99.1	98.2	98.8
			Reference	99.1	100.0	99.3
Guyana	2019	MICS	Indigenous	48.2	41.8	43.7
			African descent	75.5	61.8	73.5
			Reference	71.2	60.2	69.1
Honduras	2019	MICS	Indigenous	91.6	79.3	83.8
			African descent	86.5	90.9	87.5
			Reference	93.3	70.6	85.1
Mexico	2015	MICS	Indigenous	91.1	72.6	76.1
			Reference	90.5	85.3	88.4
Paraguay	2016	MICS	Indigenous	92.6	77.1	84.6
			Reference	96.6	87.9	95.5
Peru	2019	DHS	Indigenous	87.5	80.3	82.8
			African descent	-	54.3	54.3
			Reference	92.0	86.4	90.6
Suriname	2018	MICS	Indigenous	60.0	74.3	67.7
			African descent	75.8	75.4	75.6
			Reference	82.4	72.7	79.9

References

1. Home - UNICEF MICS [Internet]. [cited 2022 Nov 3]. Available from: https://mics.unicef.org/

2. DHS. The DHS Program - Methodology [Internet]. [cited 2022 Nov 3]. Available from: https://dhsprogram.com/methodology/index.cfm

3. Corsi DJ, Neuman M, Finlay JE, Subramanian S v. Demographic and health surveys: A profile. Int J Epidemiol [Internet]. 2012; 41(6):1602–13. Available from: https://pubmed.ncbi.nlm.nih.gov/23148108/

4. Arroyave L, Saad GE, Victora CG, Barros AJD. A new content-qualified antenatal care coverage indicator: Development and validation of a score using national health surveys in low- and middle-income countries. J Glob Health. 2021; 11:1–9.

5. Dirksen J, Pinilla-Roncancio M, Wehrmeister FC, Ferreira LZ, Vidaletti LP, Kirkby K, et al. Exploring the potential for a new measure of socioeconomic deprivation status to monitor health inequality. Int J Equity Health. 2022 ;21(1):56. Available from: /pmc/articles/PMC9034442/

Supplementary methods 2: Scoping review methods on equity-informed maternal health interventions

This supplementary methods section describes the methodological approach used for the scoping review on equity-informed maternal health interventions.

Aim and objectives

The overall aim of this scoping review is to identify how equity- and intersectionality-informed approaches have been used in existing interventions to address social power relations in maternal health. The specific objectives are:

1. To identify and describe interventions (types of intervention, design) that use equity- or intersectionality-informed approaches in maternal health;
2. To assess the extent to which the PROGRESS+ social factors are considered in global maternal health interventions, by mapping the types of interventions, target populations, and outcome evaluation.

Topic of interest

We operationally defined 'equity-informed interventions' as interventions that included mechanisms of action that directly or explicitly aim to reduce inequities or promote equity in maternal health or maternal healthcare (e.g., voucher programmes for free antenatal care targeting the poor, community mobilisation or outreach targeting marginalised communities to improve healthcare access and use). We operationally defined 'intersectionality-informed interventions' as interventions that explicitly stated the application of intersectionality in designing or implementing an intervention or programme (e.g., consideration of whether the intervention was selected based on identifying a problem using an intersectional perspective such as being representative of the experiences of diverse groups of people, or whether the intervention was designed to lead to a change in power relations). The operational definition of what constitutes an 'intersectional intervention' is inclusive of interventions using non-randomised designs, implementation of new models of care, behavioural change approaches, complex interventions, and/or participatory approaches (e.g. co-design activities, workshops, sensitization training, simulation).

We included studies that focused on an intervention to reduce inequity or promote equity in maternal health or healthcare settings. We defined maternal health and healthcare settings for this review as the following:

- Pregnancy, including antenatal care
- Childbirth, including intrapartum care (including care for babies born preterm)
- Postpartum, including postnatal care up to 1 year after birth
- Care for women and families experiencing miscarriage or stillbirth
- Maternal mental health and healthcare
- Abortion and abortion care

Types of studies

We included randomised and non-randomised trials, pre-post studies (with or without a control group), interrupted time series, realist evaluations, and other designs that compare the interventions with usual care. This review will focus on quantitative evaluation of intervention studies that have clear quantitative measures of changes in inequities in maternal health outcomes of indicators of process or experience of care, or access to maternity care. Intervention studies

published in abstract form only were not eligible for inclusion. Where we identified study protocols, we forward reference searched to identify any results publications for relevance.

We included studies that focused on assessing interventions with the following characteristics:

1. Interventions that include mechanisms that directly/explicit aim to reduce inequities or promote equity in maternal health or maternal healthcare, or use intersectionality-informed intervention design.
2. Types of outcomes:
    a. Healthcare user health and well-being outcomes (including satisfaction, health related quality of life, functioning and psychological outcomes, positive childbirth experience),
    b. Healthcare access and utilization outcomes,
3. Interventions that target the following population groups or domains:
    a. Healthcare users or communities,
    b. Healthcare providers,
    c. Healthcare facilities,
    d. Health systems,
    e. Health laws or policies.

We included studies published in any language (our team has language abilities in English, French, Spanish, Portuguese, Bahasa Indonesia, and Arabic).

Types of participants and settings

We included studies with all types of participants who are healthcare users, family members of healthcare users or community members, health workers of any cadre, policy-makers, or other key stakeholders with no restrictions to sociodemographics or social identities.

We included studies conducted in any country globally. We included studies where interventions target any type of setting where maternal healthcare services are received, such as health facilities, community-based care, or home-based care.

Search methods for identifying studies

We searched Medline and CINAHL databases. We developed search strategies for each database, without any limits on language or publication date (see below). We searched all databases from inception to 23 May 2022. In addition to database searching, we reviewed the reference lists of all included studies.

Selection of studies

We collated all title and abstracts identified from different searches into one reference database (EndNote) and removed duplicates. Two review authors independently assessed each record for its potential inclusion eligibility based on predefined criteria using Covidence. We excluded references that did not meet the eligibility criteria. Then we retrieved the full text of all the studies identified as potentially relevant after the title and abstract screening. All full texts were assessed by two review authors independently using Covidence. We resolved any disagreements between two authors through discussion and consensus, or referred to a third reviewer for a final decision. A PRISMA flow diagram illustrating our search results and the process of screening and selecting studies for inclusion is included in the main text.

Language translation

For title and abstract assessment of studies published in languages that none of the review team are fluent in, we carried out initial translation through open source software (Google Translate). If the

translation indicated agreement with the inclusion criteria, or if the translation is insufficient to decide, we will ask other colleagues in our networks to assist in assessing full text for inclusion.

Data extraction and analysis

We designed a data extraction form for this review to extract data based on study setting, sample characteristics, objectives, guiding frameworks, study design, intervention design and components, data collection tools and analysis methods, study outcomes, and author conclusions.

We conducted a descriptive analysis to report data in Supplementary Table 1 regarding country, country-income level, region, study setting (urban, rural, both), and date of publication. We conducted a descriptive analysis to report data in Supplementary Table 2 regarding study design, data source (primary, secondary), duration of intervention, sample size, health topic, target population, and outcome evaluation. We reclassified the types of intervention, target population, and outcome evaluation to map each to the PROGRESS+ social factors, which we defined for this review as:

1. Place of residence
2. Race, ethnicity, culture, language
3. Occupation
4. Gender/sex
5. Religion
6. Education
7. Socioeconomic status
8. Social capital
9. Refugee/migration status
10. Age
11. Justice-involved individuals

We report the extent to which PROGRESS+ social factors were considered in the included interventions in Table 1, by number and percentage.

Description of included studies

The following studies were included in the scoping review: (1-59). Most of the 59 included studies were randomised trials (14 studies, 23.7%) or quasi-experimental studies (12 studies, 20.3%), 9 were pre/post designs (15.3%), and 9 employed other evaluation designs (15.3%). Most included studies generated primary data (47 studies, 79.7%).

The 59 studies varied in terms of the maternal health topic, with 38 studies (64.4%) focusing on access and use of maternal health services (antenatal care, childbirth care, and postnatal care), five studies (8.5%) about maternal health quality of care (respectful care, integrated maternal and newborn care, cultural safety), five studies (8.5%) about health systems and governance (mHealth for continuum of care, community mobilisation, maternity protection laws, maternity leave), four studies (6.8%) about maternal and child nutrition (undernutrition, integrated nutrition and health, first 1000 days), four studies (6.8%) about postnatal and neonatal health (preterm birth, low birthweight, breastfeeding, neonatal mortality), and three studies (5.1%) were about contraception (modern contraceptive uptake). All studies had explicit aims of improving equity through the programme and/or intervention design, most commonly via health behaviour change interventions (e.g., community health promotion, strengthening community-health facility connections), introduction of free or subsidised maternity care services, and conditional or unconditional cash transfers.

Approximately half of the included studies specifically targeted underserved or marginalised groups (equity-informed target population, 31/59 studies, 52.5%), most commonly women experiencing

poverty (17/31 studies, 54.8%), Indigenous women (3/31 studies, 9.7%), women living in rural areas (4/31 studies, 12.9%), women from ethnic minority backgrounds (2/31 studies, 6.5%), or women experiencing food insecurity (2/31 studies, 6.5%).

Almost all studies had equity-informed outcome evaluation (51/59 studies, 86.4%), either disaggregating data related to equity identifiers or calculating concentration indices. The most common dimensions of equity-informed outcome evaluation were wealth status (41/51 studies, 80.4%), education (14/51 studies, 27.5%), age (11/51 studies, 21.6%), urban or rural place of residence (10/51 studies, 19.6%), or caste (8/51 studies, 15.7%) (note: more than one equity outcome reported in 27/59 studies).

Scoping review references

1. Yelland J, Mensah F, Riggs E, McDonald E, Szwarc J, Dawson W, et al. Evaluation of systems reform in public hospitals, Victoria, Australia, to improve access to antenatal care for women of refugee background: An interrupted time series design.e1003089-.
2. Witter S, Boukhalfa C, Cresswell JA, Daou Z, Filippi V, Ganaba R, et al. Cost and impact of policies to remove and reduce fees for obstetric care in Benin, Burkina Faso, Mali and Morocco.123.
3. Willey B, Umar N, Beaumont E, Allen E, Anyanti J, Bello AB, et al. Improving maternal and newborn health services in Northeast Nigeria through a government-led partnership of stakeholders: a quasi-experimental study.e048877.
4. Warren CE, Abuya T, Kanya L, Obare F, Njuki R, Temmerman M, et al. A cross sectional comparison of postnatal care quality in facilities participating in a maternal health voucher program versus non-voucher facilities in Kenya.153-.
5. Ward VC, Weng Y, Bentley J, Carmichael SL, Mehta KM, Mahmood W, et al. Evaluation of a large-scale reproductive, maternal, newborn and child health and nutrition program in Bihar, India, through an equity lens.021011.
6. Vellakkal S, Gupta A, Khan Z, Stuckler D, Reeves A, Ebrahim S, et al. Has India's national rural health mission reduced inequities in maternal health services? A pre-post repeated cross-sectional study.79-90.
7. Spangler SA, Barry D, Sibley L. An evaluation of equitable access to a community-based maternal and newborn health program in rural Ethiopia.S101-9.
8. Schuler SR, Nanda G, Ramírez LF, Chen M. INTERACTIVE WORKSHOPS TO PROMOTE GENDER EQUITY AND FAMILY PLANNING IN RURAL COMMUNITIES OF GUATEMALA: RESULTS OF A COMMUNITY RANDOMIZED STUDY.667-86.
9. Sarmiento I, Paredes-Solís S, de Jesús García A, Maciel Paulino N, Serrano de Los Santos FR, Legorreta-Soberanis J, et al. Safe birth in cultural safety in southern Mexico: a pragmatic non-inferiority cluster-randomised controlled trial.43.
10. Sandner M, Cornelissen T, Jungmann T, Herrmann P. Evaluating the effects of a targeted home visiting program on maternal and child health outcomes.269-83.
11. Reeve C, Banfield S, Thomas A, Reeve D, Davis S. Community outreach midwifery-led model improves antenatal access in a disadvantaged population.200-6.
12. Randive B, San Sebastian M, De Costa A, Lindholm L. Inequalities in institutional delivery uptake and maternal mortality reduction in the context of cash incentive program, Janani Suraksha Yojana: results from nine states in India.1-6.
13. Quayyum Z, Khan MN, Quayyum T, Nasreen HE, Chowdhury M, Ensor T. "Can community level interventions have an impact on equity and utilization of maternal health care" - evidence from rural Bangladesh.22.
14. Priedeman Skiles M, Curtis SL, Basinga P, Angeles G. An equity analysis of performance-based financing in Rwanda: are services reaching the poorest women? :825-37.

15. Powell-Jackson T, Neupane BD, Tiwari S, Tumbahangphe K, Manandhar D, Costello AM. The impact of Nepal's national incentive programme to promote safe delivery in the district of Makwanpur.221-49.
16. Paul S, James KS. Universalisation versus targeting in maternal and child health care provisioning: Evidence from India.100502.
17. Parmar D, Banerjee A. How do supply- and demand-side interventions influence equity in healthcare utilisation? Evidence from maternal healthcare in Senegal.112582.
18. Paredes KP. Inequality in the use of maternal and child health services in the Philippines: do pro-poor health policies result in more equitable use of services? :181.
19. Mwase T, Lohmann J, Hamadou S, Brenner S, Somda SMA, Hien H, et al. Can Combining Performance-Based Financing With Equity Measures Result in Greater Equity in Utilization of Maternal Care Services? Evidence From Burkina Faso.308-22.
20. Mohan D, Scott K, Shah N, Bashingwa JJH, Chakraborty A, Ummer O, et al. Can health information through mobile phones close the divide in health behaviours among the marginalised? An equity analysis of Kilkari in Madhya Pradesh, India.
21. McDougal L, Atmavilas Y, Hay K, Silverman JG, Tarigopula UK, Raj A. Making the continuum of care work for mothers and infants: Does gender equity matter? Findings from a quasi-experimental study in Bihar, India.e0171002.
22. Målqvist M, Hoa DP, Persson L, Ekholm Selling K. Effect of Facilitation of Local Stakeholder Groups on Equity in Neonatal Survival; Results from the NeoKIP Trial in Northern Vietnam.e0145510.
23. Malik SM, Ashraf N. Equity in the use of public services for mother and newborn child health care in Pakistan: a utilization incidence analysis.120.
24. Maldonado LY, Songok JJ, Snelgrove JW, Ochieng CB, Chelagat S, Ikemeri JE, et al. Promoting positive maternal, newborn, and child health behaviors through a group-based health education and microfinance program: a prospective matched cohort study in western Kenya.1-14.
25. Lewkowitz AK, López JD, Carter EB, Duckham H, Strickland T, Macones GA, et al. Impact of a novel smartphone application on low-income, first-time mothers' breastfeeding rates: a randomized controlled trial.100143.
26. Kuwawenaruwa A, Ramsey K, Binyaruka P, Baraka J, Manzi F, Borghi J. Implementation and effectiveness of free health insurance for the poor pregnant women in Tanzania: A mixed methods evaluation.17-25.
27. Kusuma D, Cohen J, McConnell M, Berman P. Can cash transfers improve determinants of maternal mortality? Evidence from the household and community programs in Indonesia.10-20.
28. Keya KT, Bellows B, Rob U, Warren C. Improving Access to Delivery Care and Reducing the Equity Gap Through Voucher Program in Bangladesh: Evidence From Difference-in-Differences Analysis.137-45.
29. Karim AM, Tamire A, Medhanyie AA, Betemariam W. Changes in equity of maternal, newborn, and child health care practices in 115 districts of rural Ethiopia: implications for the health extension program.238.
30. Jahn JL, Simes JT. Prenatal healthcare after sentencing reform: heterogeneous effects for prenatal healthcare access and equity.954.
31. Hewitt B, Strazdins L, Martin B. The benefits of paid maternity leave for mothers' post-partum health and wellbeing: Evidence from an Australian evaluation.97-105.
32. Hazra A, Atmavilas Y, Hay K, Saggurti N, Verma RK, Ahmad J, et al. Effects of health behaviour change intervention through women's self-help groups on maternal and newborn health practices and related inequalities in rural india: A quasi-experimental study.100198.
33. Harris-Fry HA, Paudel P, Harrisson T, Shrestha N, Jha S, Beard BJ, et al. Participatory Women's Groups with Cash Transfers Can Increase Dietary Diversity and Micronutrient Adequacy during Pregnancy, whereas Women's Groups with Food Transfers Can Increase Equity in Intrahousehold Energy Allocation.1472-83.

34. Harris-Fry H, Saville NM, Paudel P, Manandhar DS, Cortina-Borja M, Skordis J. Relative power: Explaining the effects of food and cash transfers on allocative behaviour in rural Nepalese households.102784.
35. Haider MR, Rahman MM, Moinuddin M, Rahman AE, Ahmed S, Khan MM. Impact of maternal and neonatal health initiatives on inequity in maternal health care utilization in Bangladesh.e0181408.
36. Gullo S, Kuhlmann AS, Galavotti C, Msiska T, Nathan Marti C, Hastings P. Creating spaces for dialogue: a cluster-randomized evaluation of CARE's Community Score Card on health governance outcomes.858.
37. George AS, Mohan D, Gupta J, LeFevre AE, Balakrishnan S, Ved R, et al. Can community action improve equity for maternal health and how does it do so? Research findings from Gujarat, India.125.
38. Garchitorena A, Miller AC, Cordier LF, Ramananjato R, Rabeza VR, Murray M, et al. In Madagascar, Use Of Health Care Services Increased When Fees Were Removed: Lessons For Universal Health Coverage.1443-51.
39. Ganaba R, Ilboudo PGC, Cresswell JA, Yaogo M, Diallo CO, Richard F, et al. The obstetric care subsidy policy in Burkina Faso: what are the effects after five years of implementation? Findings of a complex evaluation.84.
40. Engineer CY, Dale E, Agarwal A, Alonge O, Edward A, Gupta S, et al. Effectiveness of a pay-for-performance intervention to improve maternal and child health services in Afghanistan: a cluster-randomized trial.451-9.
41. Dzakpasu S, Soremekun S, Manu A, Ten Asbroek G, Tawiah C, Hurt L, et al. Impact of free delivery care on health facility delivery and insurance coverage in Ghana's Brong Ahafo Region.e49430.
42. Dennis ML, Benova L, Goodman C, Barasa E, Abuya T, Campbell OMR. Examining user fee reductions in public primary healthcare facilities in Kenya, 1997-2012: effects on the use and content of antenatal care.35.
43. Delgado I, Cabieses B, Apablaza M, Castillo C, Aguilera X, Matute I, et al. Evaluation of the effectiveness and equity of the maternity protection reform in Chile from 2000 to 2015.e0221150.
44. De Allegri M, Ridde V, Louis VR, Sarker M, Tiendrebéogo J, Yé M, et al. The impact of targeted subsidies for facility-based delivery on access to care and equity - evidence from a population-based study in rural Burkina Faso.439-53.
45. Davidson C, Denning S, Thorp K, Tyer-Viola L, Belfort M, Sangi-Haghpeykar H, et al. Examining the effect of quality improvement initiatives on decreasing racial disparities in maternal morbidity.
46. Cunningham K, Singh A, Pandey Rana P, Brye L, Alayon S, Lapping K, et al. Suaahara in Nepal: An at-scale, multi-sectoral nutrition program influences knowledge and practices while enhancing equity.
47. Comrie-Thomson L, Webb K, Patel D, Wata P, Kapamurandu Z, Mushavi A, et al. Engaging women and men in the gender-synchronised, community-based Mbereko+Men intervention to improve maternal mental health and perinatal care-seeking in Manicaland, Zimbabwe: A cluster-randomised controlled pragmatic trial.04042.
48. Choudhary TS, Mazumder S, Haaland Ø A, Taneja S, Bahl R, Martines J, et al. Health equity impact of community-initiated kangaroo mother care: a randomized controlled trial.263.
49. Callaghan-Koru JA, Nonyane BA, Guenther T, Sitrin D, Ligowe R, Chimbalanga E, et al. Contribution of community-based newborn health promotion to reducing inequities in healthy newborn care practices and knowledge: evidence of improvement from a three-district pilot program in Malawi.1052.
50. Brownell M, Nickel NC, Chartier M, Enns JE, Chateau D, Sarkar J, et al. An Unconditional Prenatal Income Supplement Reduces Population Inequities In Birth Outcomes.447-55.

51. Brazier E, Andrzejewski C, Perkins ME, Themmen EM, Knight RJ, Bassane B. Improving poor women's access to maternity care: Findings from a primary care intervention in Burkina Faso.682-90.
52. Beck D, Veliz PT, Munro-Kramer M, Boyd C, Sakala I, Chiboola N, et al. Exploring change over time in community mobilization domains: results from a maternity waiting home intervention in rural Zambia.228.
53. Baqui AH, Rosecrans AM, Williams EK, Agrawal PK, Ahmed S, Darmstadt GL, et al. NGO facilitation of a government community-based maternal and neonatal health programme in rural India: improvements in equity.234-43.
54. Balakrishnan R, Gopichandran V, Chaturvedi S, Chatterjee R, Mahapatra T, Chaudhuri I. Continuum of Care Services for Maternal and Child Health using mobile technology - a health system strengthening strategy in low and middle income countries.84.
55. Bajracharya A, Veasnakiry L, Rathavy T, Bellows B. Increasing Uptake of Long-Acting Reversible Contraceptives in Cambodia Through a Voucher Program: Evidence From a Difference-in-Differences Analysis.S109.
56. Amudhan S, Mani K, Rai SK, Pandav CS, Krishnan A. Effectiveness of demand and supply side interventions in promoting institutional deliveries--a quasi-experimental trial from rural north India.769-80.
57. Ali M, Azmat SK, Hamza HB, Rahman MM, Hameed W. Are family planning vouchers effective in increasing use, improving equity and reaching the underserved? An evaluation of a voucher program in Pakistan.200.
58. Ahmed S, Khan MM. Is demand-side financing equity enhancing? Lessons from a maternal health voucher scheme in Bangladesh.1704-10.
59. Abuya T, Ndwiga C, Ritter J, Kanya L, Bellows B, Binkin N, et al. The effect of a multi-component intervention on disrespect and abuse during childbirth in Kenya.224.

Supplementary Table 1 – Scoping review summary of characteristics of included studies

Characteristics	n (%)<br>n=59 studies
**Country[1] (n=62)**	
Afghanistan	1 (1.6%)
Australia	3 (4.8%)
Bangladesh	4 (6.5%)
Benin	1 (1.6%)
Burkina Faso	5 (8.1%)
Cambodia	1 (1.6%)
Canada	1 (1.6%)
Chile	1 (1.6%)
Ethiopia	2 (3.2%)
Germany	1 (1.6%)
Ghana	1 (1.6%)
Guatemala	1 (1.6%)
India	12 (19.4%)
Indonesia	1 (1.6%)
Kenya	4 (6.5%)
Madagascar	1 (1.6%)
Malawi	2 (3.2%)
Mali	1 (1.6%)
Mexico	1 (1.6%)
Morocco	1 (1.6%)
Nepal	4 (6.5%)
Nigeria	1 (1.6%)
Pakistan	2 (3.2%)
Philippines	1 (1.6%)
Rwanda	1 (1.6%)
Senegal	1 (1.6%)
Tanzania	1 (1.6%)
USA	3 (4.8%)
Vietnam	1 (1.6%)
Zambia	1 (1.6%)
Zimbabwe	1 (1.6%)
**Country-income level[2] (n=62)**	
Low	19 (30.6%)
Lower-middle	33 (53.2%)
Upper-middle	1 (1.6%)
High	9 (14.5%)
**Region**	
African	21 (35.6%)
Americas	7 (11.9%)
Eastern Mediterranean	1 (1.7%)
European	1 (1.7%)
Southeast Asian	23 (39.0%)
Western Pacific	6 (10.2%)
**Study setting**	
Urban	2 (3.4%)
Rural	24 (40.7%)
Both urban and rural	17 (28.8%)
Not specified	16 (27.1%)
**Date of publication**	
Before 2010	3 (5.1%)
2010-2014	9 (15.3%)
2015-2019	31 (52.5%)
2020-2022	16 (27.1%)

[1] One multi country study was conducted in Burkina Faso, Benin, Mali and Morocco (Witter 2016). [2]; One multi country study was conducted in both low- and lower-middle income countries (Witter 2016).

Supplementary Table 2 - Scoping review characteristics of included studies at the study-level

This table presents the characteristics of the 59 studies included in the scoping review component at a study level.

Author (year)	Title	Country	Location	Type of region	Income level (at publication)	WHO Region[1]	Health topic
**Abuya (2015)**	The effect of a multi-component intervention on disrespect and abuse during childbirth in Kenya	Kenya	Kisumu, Kiambu, Nyandarua, Uasin Gishu, and a slum area in Nairobi	Urban and Rural	Lower middle income	AFRO	Maternal health - quality of care (respectful care)
**Ahmed (2011)**	Is demand-side financing equity enhancing? Lessons from a maternal health voucher scheme in Bangladesh	Bangladesh	Sarishabari subdistrict	Rural	Low income	SEARO	Maternal health - access and service use (antenatal care, childbirth, and postnatal care)
**Ali (2019)**	Are family planning vouchers effective in increasing use, improving equity and reaching the underserved? An evaluation of a voucher program in Pakistan	Pakistan	Chakwal and Bhakkar districts, Punjab province	Urban and Rural	Lower middle income	SEARO	Contraception (modern contraceptive uptake)
**Amudhan (2013)**	Effectiveness of demand and supply side interventions in promoting institutional deliveries – a quasi-experimental trial from rural north India	India	Ballabgarh town and Dayalpur and Chhainsa	Rural	Lower middle income	SEARO	Maternal health - access and service use (childbirth)
**Bajracharya (2016)**	Increasing uptake of long-acting reversible contraceptives in Cambodia through a voucher program: evidence from a difference-in-differences analysis	Cambodia	Kampong Thom, Kampot, and Prey Veng provinces	Not specified	Lower middle income	WPRO	Contraception (modern contraceptive uptake)
**Balakrishnan (2016)**	Continuum of Care Services for Maternal and Child Health using mobile technology – a health system strengthening strategy in low and middle income countries	India	Saharsa district of Bihar	Rural	Lower middle income	SEARO	Health systems and governance (mHealth for maternal health services)
**Baqui (2008)**	NGO facilitation of a government community-based maternal and neonatal health programme in rural India: improvements in equity	India	Barbanki and Unnao districts, Uttar Pradesh	Rural	Lower middle income	SEARO	Maternal and child nutrition (Integrated Nutrition and Health Programme)
**Beck (2021)**	Exploring change over time in community mobilization domains: results from a maternity waiting home intervention in rural Zambia	Zambia	Mansa and Lundazi districts	Rural	Lower middle income	AFRO	Health systems and governance (community mobilization)
**Brazier (2009)**	Improving poor women's access to maternity care: findings from a primary care intervention in Burkina Faso	Burkina Faso	Ouargaye and Diapaga districts	Urban and Rural	Low income	AFRO	Maternal health - access and service use (availability and accessibility of quality care)
**Brownell (2018)**	An unconditional prenatal income supplement reduces population inequities in birth outcomes	Canada	Manitoba province	Not specified	High income	PAHO	Postnatal and neonatal health (preterm birth, and low-birthweight)

Author (year)	Title	Country	Location	Type of region	Income level (at publication)	WHO Region[1]	Health topic
**Callaghan-Koru (2013)**	Contribution of community-based newborn health promotion to reducing inequities in healthy newborn care practices and knowledge: evidence of improvement from a three-district pilot program in Malawi	Malawi	Thyolo, Dowa, and Chitipa districts	Rural	Low income	AFRO	Maternal health - quality of care (integrated maternal and newborn care and community-based maternal and newborn care)
**Choudhary (2021)**	Health equity impact of community-initiated kangaroo mother care: a randomized controlled trial	India	Faridabad and Palwal district, Haryana	Not specified	Lower middle income	SEARO	Postnatal and neonatal health (low-birthweight)
**Comrie-Thomson (2022)**	Engaging women and men in the gender-synchronised, community-based Mbereko+Men intervention to improve maternal mental health and perinatal care-seeking in Manicaland, Zimbabwe: a cluster-randomised controlled pragmatic trial	Zimbabwe	Mutasa district	Rural	Lower middle income	AFRO	Maternal health - access and service use (postnatal depression and male engagement)
**Cunningham (2017)**	Suaahara in Nepal: an at-scale, multi-sectoral nutrition program influences knowledge and practices while enhancing equity	Nepal	Not specified (eight rural districts)	Rural	Low income	SEARO	Maternal and child nutrition (first 1,000 days)
**Davidson (2022)**	Examining the effect of quality improvement initiatives on decreasing racial disparities in maternal morbidity	USA	Texas	Not specified	High income	PAHO	Maternal health - quality of care (obstetric haemorrhage)
**DeAllegri (2012)**	The impact of targeted subsidies for facility-based delivery on access to care and equity – evidence from a population-based study in rural Burkina Faso	Burkina Faso	Nouna district	Rural	Low income	AFRO	Maternal health - access and service use (childbirth)
**Delgado (2019)**	Evaluation of the effectiveness and equity of the maternity protection reform in Chile from 2000 to 2015	Chile	Nationwide	Urban and Rural	High income	PAHO	Health systems and governance (maternity protection law for childcare and women's paid work)
**Dennis (2020)**	Examining user fee reductions in public primary healthcare facilities in Kenya, 1997–2012: effects on the use and content of antenatal care	Kenya	Nationwide	Urban and Rural	Lower middle income	AFRO	Maternal health - access and service use (antenatal care)
**Dzakpasu (2012)**	Impact of free delivery care on health facility delivery and insurance coverage in Ghana's Brong Ahafo Region	Ghana	Brong Ahafo region	Rural	Lower middle income	AFRO	Maternal health - access and service use (childbirth)
**Engineer (2016)**	Effectiveness of a pay-for-performance intervention to improve maternal and child health services in Afghanistan: a cluster-randomized trial	Afghanistan	Not specified (11 provinces)	Not specified	Low income	EMRO	Maternal health - access and service use (antenatal and postnatal care)

Author (year)	Title	Country	Location	Type of region	Income level (at publication)	WHO Region[1]	Health topic
**Ganaba (2016)**	The obstetric care subsidy policy in Burkina Faso: what are the effects after five years of implementation? Findings of a complex evaluation	Burkina Faso	Bogandé, Houndé, Orodara, Yako, Banfora, Gaoua districts	Not specified	Low income	AFRO	Maternal health - access and service use (childbirth)
**Garchitorena (2017)**	In Madagascar, use of health care services increased when fees were removed: lessons for universal health coverage	Madagascar	Ifanadiana district	Rural	Low income	AFRO	Maternal health - access and service use (antenatal, childbirth, and postnatal care)
**George (2018)**	Can community action improve equity for maternal health and how does it do so? Research findings from Gujarat, India	India	Dahod, Panchmahal and Anand districts, Gujarat	Urban and Rural	Lower middle income	SEARO	Maternal health - access and service use (antenatal and childbirth care)
**Gullo (2018)**	Creating spaces for dialogue: a cluster randomized evaluation of CARE's Community Score Card on health governance outcomes	Malawi	Ntcheu district	Not specified	Low income	AFRO	Health systems and governance (power sharing, equity)
**Haider (2017)**	Impact of maternal and neonatal health initiatives on inequity in maternal health care utilization in Bangladesh	Bangladesh	Maulvibazar, Jamalpur, Narail and Thakurgaon districts	Urban and Rural	Lower middle income	SEARO	Maternal health - access and service use (antenatal, childbirth, and postnatal care)
**Harris-Fry (2018)**	Participatory women's groups with cash transfers can increase dietary diversity and micronutrient adequacy during pregnancy, whereas women's groups with food transfers can increase equity in intrahousehold energy allocation	Nepal	Dhanusha and Mahottari districts	Rural	Low income	SEARO	Maternal and child nutrition (maternal undernutrition)
**Harris-Fry (2022)**	Relative power: explaining the effects of food and cash transfers on allocative behaviour in rural Nepalese households	Nepal	Dhanusha and Mahottari districts	Rural	Lower middle income	SEARO	Maternal and child nutrition (maternal undernutrition)
**Hazra (2020)**	Effects of health behaviour change intervention through women's self-help groups on maternal and newborn health practices and related inequalities in rural India: a quasi-experimental study	India	Uttar Pradesh	Rural	Lower middle income	SEARO	Maternal health - access and service use (antenatal care, childbirth, postnatal care, and contraception)
**Hewitt (2017)**	The benefits of paid maternity leave for mothers' post-partum health and wellbeing: evidence from an Australian evaluation	Australia	Nationwide	Not specified	High income	WPRO	Health systems and governance (paid maternity leave)
**Jahn (2022)**	Prenatal healthcare after sentencing reform: heterogeneous effects for prenatal healthcare access and equity	USA	Pennsylvania	Urban and Rural	High income	PAHO	Maternal health - access and service use (justice reform and antenatal care)
**Karim (2015)**	Changes in equity of maternal, newborn, and child health care practices in 115 districts of rural Ethiopia: implications for the health extension program	Ethiopia	Amhara, Oromia, Tigray, and Southern Nations regions	Rural	Low income	AFRO	Maternal health - access and service use (antenatal care, childbirth, postnatal care, and contraception)

Author (year)	Title	Country	Location	Type of region	Income level (at publication)	WHO Region[1]	Health topic
**Keya (2018)**	Improving access to delivery care and reducing the equity gap through voucher program in Bangladesh: evidence from difference-in-differences analysis	Bangladesh	Not specified (11 upazilas)	Rural	Lower middle income	SEARO	Maternal health - access and service use (childbirth)
**Kusuma (2016)**	Can cash transfers improve determinants of maternal mortality? Evidence from the household and community programs in Indonesia	Indonesia	West Java, East Java, North Sulawesi, Gorontalo, East Nusa Tenggara and Jakarta provinces	Urban and Rural	Lower middle income	SEARO	Maternal health - access and service use (antenatal, childbirth, and postnatal care)
**Kuwawenaruwa (2019)**	Implementation and effectiveness of free health insurance for the poor pregnant women in Tanzania: a mixed methods evaluation	Tanzania	Mbarali in Mbeya region and Kilolo in Iringa region	Not specified	Lower middle income	AFRO	Maternal health - access and service use (antenatal, childbirth, and postnatal care)
**Lewkowitz (2020)**	Impact of a novel smartphone application on low-income, first-time mothers' breastfeeding rates: a randomized controlled trial	USA	Midwest	Urban	High income	PAHO	Postnatal and neonatal health (breastfeeding)
**Maldonado (2020)**	Promoting positive maternal, newborn, and child health behaviors through a groupbased health education and microfinance program: a prospective matched cohort study in western Kenya	Kenya	Bunyala, Busia, Western Province	Rural	Lower middle income	AFRO	Maternal health - access and service use (antenatal care, childbirth, postnatal care, and contraception)
**Malik (2016)**	Equity in the use of public services for mother and newborn child health care in Pakistan: a utilization incidence analysis	Pakistan	Nationwide	Urban and Rural	Lower middle income	SEARO	Maternal health - access and service use (antenatal, childbirth, and postnatal care)
**Målqvist (2015)**	Effect of facilitation of local stakeholder groups on equity in neonatal survival; results from the NeoKIP trial in Northern Vietnam	Vietnam	Quang Ninh province	Not specified	Lower middle income	WPRO	Postnatal and neonatal health (neonatal mortality)
**McDougal (2017)**	Making the continuum of care work for mothers and infants: does gender equity matter? findings from a quasi-experimental study in Bihar, India	India	Bihar	Not specified	Lower middle income	SEARO	Maternal health - access and service use (antenatal, childbirth, and postnatal care)
**Mohan (2021)**	Can health information through mobile phones close the divide in health behaviours among the marginalised? An equity analysis of Kilkari in Madhya Pradesh, India	India	Rajgarh, Rewa, Hoshangabad, Mandsaur states	Not specified	Lower middle income	SEARO	Maternal health - access and service use (childbirth and contraception)
**Mwase (2022)**	Can combining performance-based financing with equity measures result in greater equity in utilization of maternal care services? Evidence from Burkina Faso	Burkina Faso	Boucle du Mouhoun, Centre-Nord, Centre-Ouest, Nord, Sud-Ouest, Centre-Est regions	Rural	Low income	AFRO	Maternal health - access and service use (antenatal, childbirth, and postnatal care)

Author (year)	Title	Country	Location	Type of region	Income level (at publication)	WHO Region[1]	Health topic
**Paredes (2016)**	Inequality in the use of maternal and child health services in the Philippines: do pro-poor health policies result in more equitable use of services?	Philippines	Nationwide	Urban and Rural	Lower middle income	WPRO	Maternal health - access and service use (antenatal care, childbirth, caesarean section)
**Parmar (2019)**	How do supply- and demand-side interventions influence equity in healthcare utilisation? Evidence from maternal healthcare in Senegal	Senegal	Nationwide	Urban and Rural	Lower middle income	AFRO	Maternal health - access and service use (childbirth)
**Paul (2019)**	Universalisation versus targeting in maternal and child health care provisioning: evidence from India	India	Assam, Bihar, Chhattisgarh, Jharkhand, Jammu and Kashmir, Madhya Pradesh, Odisha, Rajasthan, Uttarakhand, Uttar Pradesh, Arunachal Pradesh, Manipur, Mizoram, Meghalaya, Nagaland, Sikkim, Tripura, Kerala, Tamil Nadu, Andhra Pradesh, Karnataka, Goa, West Bengal, Punjab, Haryana, Gujarat, Himachal Pradesh, Maharashtra, and Delhi states	Urban and Rural	Lower middle income	SEARO	Maternal health - access and service use (antenatal, childbirth, and postnatal care)
**Powell-Jackson (2009)**	The impact of Nepal's national incentive programme to promote safe delivery in the District of Makwanpur	Nepal	Makwanpur district	Rural	Low income	SEARO	Maternal health - access and service use (antenatal care and childbirth)
**Priedeman Skiles (2013)**	An equity analysis of performance-based financing in Rwanda: are services reaching the poorest women?	Rwanda	Not specified (19 districts)	Urban and Rural	Low income	AFRO	Maternal health - access and service use (antenatal care, childbirth, and contraception)
**Quayyum (2013)**	"Can community level interventions have an impact on equity and utilization of maternal health care" – Evidence from rural Bangladesh	Bangladesh	Gaibandha, Rangpur, Mymensingh Netrokona and Naogaon districts	Rural	Low income	SEARO	Maternal health - access and service use (antenatal, childbirth, and postnatal care)
**Randive (2014)**	Inequalities in institutional delivery uptake and maternal mortality reduction in the context of cash incentive program, Janani Suraksha Yojana: results from nine states in India	India	Rajasthan, Madhya Pradesh, Chhattisgarh, Bihar, Jharkhand, Uttar Pradesh, Uttarakhand, Orissa and Assam states	Not specified	Lower middle income	SEARO	Maternal health - access and service use (childbirth)

Author (year)	Title	Country	Location	Type of region	Income level (at publication)	WHO Region[1]	Health topic
**Reeve (2016)**	Community outreach midwifery-led model improves antenatal access in a disadvantaged population	Australia	Fitzroy Valley of Western Australia	Not specified	High income	WPRO	Maternal health - access and service use (antenatal care and childbirth)
**Sandner (2018)**	Evaluating the effects of a targeted home visiting program on maternal and child health outcomes	Germany	Lower Saxony, Bremen and Saxony states	Urban and Rural	High income	EURO	Maternal health - access and service use (antenatal and postnatal care)
**Sarmiento (2022)**	Safe birth in cultural safety in southern Mexico: a pragmatic non-inferiority cluster-randomised controlled trial	Mexico	Guerrero State	Rural	Upper middle income	PAHO	Maternal health – quality of care (Indigenous maternal health and cultural safety)
**Schuler (2015)**	Interactive workshops to promote gender equity and family planning in rural communities of Guatemala: results of a community randomized study	Guatemala	Sacatepéquez, Chimaltenango, Sololá, Huehuetenango and San Marco	Rural	Lower middle income	PAHO	Contraception (gender equity)
**Spangler (2014)**	An evaluation of equitable access to a community-based maternal and newborn health program in rural Ethiopia	Ethiopia	Amhara and Oromiya regions	Rural	Low income	AFRO	Maternal health – quality of care (childbirth and postnatal care)
**Vellakkal (2017)**	Has India's national rural health mission reduced inequities in maternal health services? A pre-post repeated cross-sectional study	India	15 states	Urban and Rural	Lower middle income	SEARO	Maternal health - access and service use (antenatal care and childbirth)
**Ward (2020)**	Evaluation of a large-scale reproductive, maternal, newborn and child health and nutrition program in Bihar, India, through an equity lens	India	Bihar state	Not specified	Lower middle income	SEARO	Maternal health - access and service use (antenatal care, childbirth, and contraception)
**Warren (2015)**	A cross sectional comparison of postnatal care quality in facilities participating in a maternal health voucher program versus non-voucher facilities in Kenya	Kenya	Kiambu, Kisumu, Kitui, Nyandarua, Uasin Gichu, Makueni counties	Not specified	Lower middle income	AFRO	Maternal health - access and service use (postnatal care)
**Willey (2022)**	Improving maternal and newborn health services in Northeast Nigeria through a government-led partnership of stakeholders: a quasi-experimental study	Nigeria	Gombe state	Rural	Lower middle income	AFRO	Maternal health - access and service use (childbirth and caesarean section)
**Witter (2016)**	Cost and impact of policies to remove and reduce fees for obstetric care in Benin, Burkina Faso, Mali and Morocco	Benin, Burkina Faso, Mali and Morocco	Nationwide	Urban and Rural	Low income (Benin, Burkina Faso, Mali) and lower middle income (Morocco)	AFRO	Maternal health - access and service use (antenatal care, childbirth, postnatal care, and contraception)

Author (year)	Title	Country	Location	Type of region	Income level (at publication)	WHO Region[1]	Health topic
**Yelland (2020)**	Evaluation of systems reform in public hospitals, Victoria, Australia, to improve access to antenatal care for women of refugee background: an interrupted time series design	Australia	Victoria state	Urban	High income	WPRO	Maternal health - access and service use (antenatal care)

[1]AFRO = African region, PAHO = Americas region, EMRO = Eastern Mediterranean region, EURO = European region, SEARO = Southeast Asian region, WPRO = Western Pacific region

Supplementary Table 3 – Scoping review summary of intervention description and outcomes

This table presents the description of the interventions, study design and population, and narrative description of intervention results for the 59 studies included in the scoping review component, at a summary level.

Characteristics	n (%)<br>n=59 studies
**Study design**	
Randomised trial	14 (23.7%)
Quasi-experimental study	12 (20.3%)
Evaluation	9 (15.3%)
Pre/post study	9 (15.3%)
Cross-sectional study	8 (13.6%)
Time series	4 (6.8%)
Cohort study	3 (5.1%)
**Data source**	
Primary data	47 (79.7%)
Secondary data	12 (20.3%)
**Duration of intervention**	
< 1 year	2 (3.4%)
≥ 1 to < 3 years	23 (39.0%)
≥ 3 to < 5 years	14 (23.7%)
≥ 5 years	15 (25.4%)
Not specified	5 (8.5%)
**Sample size**	
≤ 999	9 (15.3%)
1,000 – 4,999	20 (33.9%)
5,000 – 9,999	8 (13.6%)
10,000 – 14,999	3 (5.1%)
15,000 – 19,999	4 (6.8%)
20,000 – 99,999	8 (13.6%)
≥ 100,000	4 (6.8%)
Not specified	3 (5.1%)
**Health topic**	
Maternal health - access and service use	38 (64.4%)
Maternal health - quality of care	5 (8.5%)
Maternal and child nutrition	4 (6.8%)
Postnatal and neonatal	4 (6.8%)
Contraception	3 (5.1%)
Health systems and governance	5 (8.5%)
**Equity-informed target population**	
Yes	31 (52.5%)
No	28 (47.5%)
**Characteristics of equity-informed target population[1] (n=31)**	
Women experiencing poverty	17 (54.8%)
Indigenous women	3 (9.7%)
Women living in rural areas	4 (12.9%)
Women and men	1 (3.2%)
Women from ethnic minority backgrounds	2 (6.5%)
Women experiencing food insecurity	2 (6.5%)
Women who work	1 (3.2%)
Women of refugee backgrounds	1 (3.2%)
**Equity-informed outcome evaluation**	
Yes	51 (86.4%)
No	8 (13.6%)
**Dimensions of equity-informed outcome evaluation[2] (n=51)**	
Wealth status	41 (80.4%)
Education	14 (27.5%)
Age	11 (21.6%)

Place of residence (urban/rural)	10 (19.6%)
Caste	8 (15.7%)
Employment status	5 (9.8%)
Gender	4 (7.8%)
Race/ethnicity	3 (5.9%)
Geographical accessibility to health facilities	3 (5.9%)
Literacy	2 (3.9%)
Religion	2 (3.9%)
Indigenous status	1 (2.0%)
Refugee status	1 (2.0%)

[1] 31/59 studies reported equity-informed target population

[2] There is more than one equity-informed outcome evaluated in 27/59 studies.

Supplementary Table 4 – Scoping review intervention description and outcomes at the study-level

This table presents the description of the interventions, study design and population, and narrative description of intervention results for the 59 studies included in the scoping review component, at a study level.

Authors (year)	Description of intervention	Study design	Study population	Sample size	Timing and duration of intervention	Equity-informed design	Equity-informed target population	Equity-informed outcome evaluation	Narrative description of intervention results
**Abuya (2015)**	Multi-component intervention to improve respectful care during childbirth, including policy dialogue, training, quality improvement teams, counselling, mentorship, maternity open days, and community workshops.	Pre/post study (mixed-methods)	Interviews: women who gave birth in prior 24-48 hours; observation: women in labour	2,569 (overall) 641 (baseline interviews) 677 (baseline observations) 728 (endline interviews) 523 (endline observations)	June 2011 to February 2014 (32 months)	Yes	No - all women	Yes - wealth status, age	The intervention had mixed success, with reductions in feeling disrespected, physical abuse, violation of privacy, verbal abuse, and detention in health facility, but no change in violations of privacy or abandonment.
**Ahmed (2011)**	Two types of voucher schemes for pregnant women to improve use of antenatal care, skilled birth attendance, childbirth in a health facility, and postnatal care: 1) universal programme for all women, and 2) targeted programme for women experiencing poverty.	Evaluation (process evaluation)	Women who had a live birth within last 12 months	3,600 (overall) 600 (intervention) 3000 (control)	April 2007 to June 2008 (14 months)	Yes	Yes - all women, and poor women	Yes - wealth status	The intervention was successful with use rates improving for antenatal care, skilled birth attendance, childbirth in a health facility and postnatal care, and rich-to-poor ratios are significantly lower in intervention areas suggesting a positive impact on equity.
**Ali (2019)**	Free, single-purpose voucher to increase access, uptake, and equity in modern contraceptive use among women experiencing poverty.	Quasi-experimental study (pre/post)	Women aged 18-49 years old from lowest two wealth quintiles	5,890 (overall) 3,276 (baseline) 2,614 (endline)	May 2012 to March 2015 (34 months)	Yes	Yes - poor women	Yes - wealth status	The intervention was successful in increasing ever use of contraception, current contraception user, and modern contraceptive method user, and reduced inequities between poorer and richer women.
**Amudhan (2013)**	Free intrapartum care services (24 hours per day, 7 days per week) and Janani Suraksha Yojana (JSY) voucher scheme for women experiencing poverty.	Quasi-experimental study (before and after with multiple control groups)	Women who gave birth during study period	3,276 (overall) 1,963 (baseline) 5,833 (endline)	April 2006 to March 2010 (47 months)	Yes	Yes - poor women	Yes - wealth status, geographical accessibility	The intervention had varying levels of success in increasing rates of childbirth in health facilities depending on the combination of JSY and primary healthcare services, however, there are clear trends in increasing rates of childbirth in health facilities and the largest increases in the most disadvantaged groups.
**Bajracharya (2016)**	Reproductive Health Voucher Program to increase poor women's access to maternal and	Quasi-experimental study	Married women not currently pregnant	1,936 (overall) 961	2011 to 2013 (24 months)	Yes	Yes - poor women	Yes - wealth status, age,	The intervention had mixed success, with a significant increase in use of long-acting reversible contraceptives and permanent

Authors (year)	Description of intervention	Study design	Study population	Sample size	Timing and duration of intervention	Equity-informed design	Equity-informed target population	Equity-informed outcome evaluation	Narrative description of intervention results
	reproductive health services and uptake and expand choice of contraceptive methods.			(intervention) 975 (control)				education, employment	contraception in the intervention areas, but no change in use of modern contraceptives.
**Balakrishnan (2016)**	Programme to introduce Continuum of Care Services (Maternal and Child) for case management for frontline community health workers to improve pregnancy registration, antenatal care, birth preparedness, childbirth, postnatal care, breastfeeding, immunization, and growth monitoring.	Evaluation (implementation project)	Pregnant women	19,880 (overall) 19,880 (baseline) Not specified (endline)	June 2012 to March 2015 (33 months)	Yes	No - all women	Yes - caste	The intervention had mixed success, with a significant increase on increasing childbirth in health facilities, but no impact on 3 antenatal care visits or 1 postnatal care visit. There was equitable distribution of intervention effects comparing 'all population' to Scheduled Castes and Scheduled Tribes.
**Baqui (2008)**	Home visitation by community-based workers during antenatal and postnatal periods for promoting use of maternal health services.	Quasi-experimental study	Women who had a live or stillbirth	28,778 (overall) 16,568 (baseline) 12,210 (endline)	January 2003 to March 2006 (38 months)	Yes	No - all women	Yes - wealth status	The intervention was successful in increasing antenatal and postnatal home visits, ≥1 antenatal care visit, and skilled birth attendance. The intervention had a positive impact on equity, with concentration indices decreasing from baseline to endline for all four outcomes, meaning that coverage in all wealth quintiles increased and inequities decreased.
**Beck (2021)**	Community mobilisation as part of a maternity waiting homes intervention. The community mobilization includes training for Safe Motherhood Action Group members on how to conduct community meetings, community meetings with traditional leaders and forming governance committees.	Evaluation (secondary analysis of longitudinal intervention)	- Women ≥15 years with babies aged <1 year old at baseline; community members; health workers	1,202 (overall) 286 (baseline - intervention) 366 (endline - intervention) 267 (baseline - control) 283 (endline - control)	May 2015 to July 2018 (38 months)	Yes	No - all women, community members and health workers	Yes - age, education	The intervention had mixed success, with community mobilisation improving community governance, social cohesion, self-efficacy, and power in relationships, but no impact on collective efficacy. This suggests community mobilisation may impact in different ways for different community members, with the most marginalised receiving the least benefit.
**Brazier (2009)**	Intervention aimed to increase access to skilled maternity care by improving training for maternity care providers, addressing gaps in essential obstetric equipment and supplies, strengthening referral	Pre/post study	Women of reproductive age and their co-resident husbands	6,021 (overall) 2,489 (baseline) 3,532 (endline)	2003 to 2006 (36 months)	Yes	Yes - poor women	Yes - wealth status	The intervention had mixed success, with a significant increase in rates of skilled birth attendance and childbirth in a health facility, but had mixed effects on equity, with rich-to-poor gaps significantly narrowing in 1 district but widening in the other district.

Authors (year)	Description of intervention	Study design	Study population	Sample size	Timing and duration of intervention	Equity-informed design	Equity-informed target population	Equity-informed outcome evaluation	Narrative description of intervention results
	system, introducing a quality assurance, improving management systems, individualised birth preparedness counselling and community-level campaign on maternal health risks and benefits of facility-based childbirth.								
**Brownell (2018)**	The Healthy Baby Prenatal Benefit is an unconditional income supplement (approx. US$64 per month) for low income pregnant women during the second and third trimesters of pregnancy.	Cohort study	All mother newborn pairs in Manitoba in the period 2003–2010	76,049 (overall)<br>10,031 (intervention group 1)<br>10,031 (intervention group 2)<br>55,987 (control)	2003 to 2010 (84 months)	Yes	Yes - poor women	Yes - wealth status, urban/ rural	The intervention had mixed success, with significant reductions in absolute and relative inequalities of low birth weight (comparing low income women in intervention group who received income supplement to control group women who were not low income and did not receive supplement) for women in both rural and urban areas. There was a significant reduction in absolute and relative inequalities for preterm birth (same intervention and control), but only for women in urban areas.
**Callaghan-Koru (2013)**	Community-based health promotion including 21 days of in-service training on integrated maternal and newborn care, training for Health Surveillance Assistants on community-based maternal and newborn care, and home visits by Health Surveillance Assistants during pregnancy and postpartum.	Pre/post study	Women 15–49 years of age who had a live birth in the last 12 months	1,803 (overall)<br>903 (baseline)<br>900 (endline)	November 2007 to June 2011 (43 months)	Yes	No - all women and newborns	Yes - wealth status	The intervention had mixed success, with significant increases to ≥1 antenatal care visit and childbirth in health facilities; however, wealth inequities significantly decreased only for childbirth in health facilities (no impact on ≥1 antenatal care visit).
**Choudhary (2021)**	Community-initiated kangaroo mother care for babies weighing 1500-2250g within 72 hours of birth.	Randomised trial (randomised controlled parallel-arm trial)	Babies 1500-2250g at birth, difficulty breathing or feeding, or less than normal movement	8,402 (overall)<br>4,480 (intervention)<br>3,922 (control)	July 2015 to October 2018 (27 months)	Yes	No - all low birthweight babies	Yes - wealth status, caste, baby sex, mother’s literacy, religion	While the original trial effectively reduced mortality to 180 days (primary analysis), the intervention reported here (equity analysis) was not successful, as there was no impact on inequities in neonatal mortality based on wealth, caste, maternal education, baby sex, or religion.
**Comrie-Thomson (2022)**	The Mbereko+Men programme engaged women and men in complementary activities to	Randomised trial (cluster randomised	Women ≥16 years who gave birth in last 6	1,405 (overall)<br>665	July 2016 to September 2017 (14 months)	Yes	Yes - all women and men	Yes - gender equitable men score	The intervention had mixed success, with a significant increase in postnatal care, first trimester antenatal care, and gender

Authors (year)	Description of intervention	Study design	Study population	Sample size	Timing and duration of intervention	Equity-informed design	Equity-informed target population	Equity-informed outcome evaluation	Narrative description of intervention results
	improve men's support for women and babies, coparents' equitable, informed health decision-making, maternal mental health, and care-seeking for maternal and newborn health.	controlled pragmatic trial)	months, and their male partners ≥16 years	(intervention) 740 (control)					equitable attitudes for men. Postpartum depression significantly reduced in both the intervention and control group. There was no impact on ≥4 antenatal care visits, childbirth in health facilities, and women's participation in decision-making.
**Cunningham (2017)**	Suaahara is an integrated nutrition, health services, family planning, water and sanitation, and agriculture intervention.	Evaluation (process evaluation of implementation project)	Women currently pregnant or with a child ≤2 years	472 (overall) 232 (intervention) 240 (control)	2012 to 2014 (24 months)	Yes	Yes – women with poor nutrition in rural areas	Yes - wealth status	The intervention was not successful, as there was no effect of the intervention on 4 antenatal care visits, childbirth in health facilities, or skilled birth attendance.
**Davidson (2022)**	The TexasAIM quality collaborative aimed to decrease racial disparities in maternal morbidity through quality improvement and patient safety initiatives, including an obstetric haemorrhage bundle and in-depth case reviews of adverse patient outcomes.	Pre/post study (quality improvement)	Women who gave birth (excluding miscarriages and ectopic pregnancies)	13,659 (overall) 4912 (baseline) 8747 (endline)	June 2018 to June 2020 (24 months)	Yes	Yes - women from ethnic minorities (African American, Hispanic)	Yes – race, ethnicity	The intervention had mixed success, with a significant decrease in severe maternal morbidity from haemorrhage, but no effect on all-cause severe maternal morbidity. Reductions in severe maternal morbidity (all cause and from haemorrhage) were only found in non-Hispanic Black women, with no change in non-Hispanic white women, non-Hispanic Asian women, or Hispanic women.
**DeAllegri (2012)**	Introduction of a new nationwide financing policy to subsidise (80%) fees associated with childbirth in health facilities.	Pre/post study	Women who gave birth between 2006 and 2010	1,934 (overall) 682 (baseline) 1252 (endline)	2006 to 2010 (48 months)	Yes	No - all women	Yes - wealth status, geographical accessibility	The intervention had mixed success, with a significant increase in childbirth in health facilities. While the wealth and geographical access (distance to health facility) inequities decreased from baseline to endline, the utilisation gap remains pro-rich and pro-short distance to health facilities.
**Delgado (2019)**	Introduction of a new national law increasing maternity leave from 12 to 24 weeks and incorporating paid paternity leave up to 5 weeks	Pre/post study	Women who gave birth to a live child	487,292 (overall)	2000 to 2015 (180 months)	Yes	No - all working women and men	Yes - wealth status, urban/ rural, age, education, Indigenous identity	The intervention had mixed success, with an overall significant increase in maternity protection coverage after reform, but no reduction of inequities (age, wealth status, place of residence, education, Indigenous identity).
**Dennis (2020)**	Introduction of a user fee reduction policy in public primary health facilities with registration charges of 10 Kenyan shillings in	Interrupted time series	Women aged 15–49 years who recently gave birth	15,230 (overall)	2003 to 2014 (132 months)	Yes	No - all women	Yes - wealth status	The intervention had mixed success, with the policy significantly associated with acceleration of increases to ≥4 antenatal care visits, early antenatal care, and good content of antenatal care. When

Authors (year)	Description of intervention	Study design	Study population	Sample size	Timing and duration of intervention	Equity-informed design	Equity-informed target population	Equity-informed outcome evaluation	Narrative description of intervention results
	dispensaries and KSh20 in health centres.								disaggregated by wealth quintiles, the policy is more beneficial to wealthier women compared to poorer women.
**Dzakpasu (2012)**	Introduction of a policy to provide free childbirth services and free national health insurance for pregnant women.	Time series	Women who recently gave birth	91,015 (overall)	January 2004 to December 2009 (71 months)	Yes	No - all women	Yes - wealth status	The intervention had mixed success, with significant increases in childbirth in health facilities following the introduction of the 2005 and 2008 policies. While inequities in childbirth in health facilities decreased over time, rich-to-poor gaps persisted at the end of the study.
**Engineer (2016)**	Introduction of pay-for-performance for maternity care providers, based on volume of work, quality of care and contraceptive prevalence rate.	Randomised trial (cluster randomised trial)	Ever-married women aged 12-49 years	1,454 (overall)<br>727 (intervention)<br>727 (control)	2010 to 2012 (24 months)	Yes	No - all women	Yes - wealth status	The intervention was not successful, as there was no effect of pay-for-performance on ≥1 antenatal care visit, skilled birth attendance, or postnatal care.
**Ganaba (2016)**	Introduction of a national subsidy for childbirth and emergency obstetric care in public healthcare facilities.	Evaluation (mixed-methods realist evaluation)	Women who recently gave birth	36,836 (overall - routine data)	Policy implementation from 2006, data collection May to December 2012	Yes	No - women who gave birth in public health facilities	Yes - wealth status, place of residence (urban/rural(	The intervention had mixed success, with significant increases in childbirth in health facilities, with greatest increases in rural areas and women in the poorest wealth quintiles, which closed but did not eliminate the rich-to-poor and urban-to-rural gaps. However, there was no effect on caesarean section rates – either overall or by wealth quintile.
**Garchitorena (2017)**	Introduction of interventions to increase healthcare access through provision of a package of free services to pregnant women and children ≤5 years, and the PIVOT programme of supply-side initiatives including infrastructure renovations, clinical support, and elimination of point-of service payments.	Cross-sectional study (with additional secondary data collection)	All pregnant women with a live birth in the past 5 years	Household survey:1,522 households<br>Health care utilisation: 986 pregnant women	January 2013 to December 2015	Yes	No - all women	Yes - wealth status, urban/ rural	The intervention had mixed success, with significant increases in the use of maternity consultations when point-of-service fees were removed for poor women. However, when fees were removed for all women, there was no impact on maternity consultations.
**George (2018)**	Evaluation of the equity effects of community action for maternal health led by non-government organizations, including group discussions, community meetings, home visits during pregnancy and postpartum,	Evaluation (mixed-methods)	Pregnant and postpartum women	2,395 (overall)	Project implementation: 2012 – 2015<br>Data collection: 2013 - 2014	Yes	No - all pregnant and postpartum women, community members and stakeholders	Yes - caste, employment, education	The intervention had mixed success, with an overall significant increase in childbirth in health facilities. However, there was no effect on equity outcomes (social vulnerability, employment, maternal education).

Authors (year)	Description of intervention	Study design	Study population	Sample size	Timing and duration of intervention	Equity-informed design	Equity-informed target population	Equity-informed outcome evaluation	Narrative description of intervention results
	monitoring of outreach antenatal services at Village Health and Nutrition Day, maternal death tracking, and dialogues with stakeholders.								
**Gullo (2018)**	Introduction of a community score card to facilitate discussion between community members and health workers on improving quality and equity of maternal health services.	Randomised trial (cluster randomised)	Women 15-49 years who recently gave birth	1,300 (overall)<br>651 (intervention)<br>649 (control)	November 2012 to December 2014 (25 months)	Yes	No - all women	Yes - wealth status	The intervention had mixed success, with a significant increase in the presence of community action groups and safe motherhood committees, but no effect on trust in health workers, power sharing, mutual responsibility, or collective efficacy.
**Haider (2017)**	Introduction of Maternal and Neonatal Health Initiatives in Bangladesh including birth planning, health system mobilisation to generate demand for maternal care services, and strengthening human resources, physical capacity, and procurement of drugs and equipment	Cross-sectional study	Women who recently gave birth	20,923 (overall)<br>13,206 (baseline)<br>7,717 (endline)	2008 to 2013 (60 months)	Yes	No - all women	Yes - wealth status, urban/ rural	The intervention had mixed success, with a significant increase in skilled birth attendance and childbirth in health facilities, but no effect on ≥4 antenatal care visits or postnatal care. Inequities in these four outcomes based on wealth and urban/rural residence all significantly reduced over time, but rich-to-poor and urban-to-rural gaps persisted.
**Harris-Fry (2018)**	Three pregnancy-focused nutrition interventions: participatory learning and action group for women, cash transfers (USD$7.50/month) to pregnant women in the participatory learning and action group, and food transfers (10kg/month to pregnant women in the participatory learning and action group.	Randomised trial (four-arm cluster randomised controlled trial)	Women in third trimester of pregnancy, living in male-headed households with in-laws	805 (overall)<br>655 (intervention)<br>150 (control)	December 2013 to October 2015 (22 months)	Yes	Yes - food insecure women (women who live with their in-laws)	Yes - Relative Dietary Energy Adequacy Ratio	The intervention had mixed success, with all 3 intervention groups having improved maternal diet and nutritional status in pregnancy, but mixed effects on relative dietary energy adequacy ratio for pregnant women compared to household heads or mothers-in-laws.
**Harris-Fry (2022)**	Three pregnancy-focused nutrition interventions: participatory learning and action group for women, cash transfers (USD$7.50/month) to pregnant women in the participatory learning and action group, and food transfers (10kg/month to pregnant women in the	Randomised trial (four-arm cluster randomised controlled trial)	Women who were in their third trimester of pregnancy, and living in male-headed households with their in-laws	800 (overall)<br>652 (intervention)<br>148 (control)	May 2015 to September 2015 (4 months)	Yes	Yes - food insecure women (women who live with their in-laws)	Yes - wealth status, absolute and relative bargaining power	The intervention had mixed success, with mixed effects of each intervention group on increasing food shares (staples, fruit and vegetables, animal) between the pregnant woman and head of household and between the pregnant woman and mother-in-law. Improvements to the pregnant woman's bargaining power mediated the effect of the cash transfer

Authors (year)	Description of intervention	Study design	Study population	Sample size	Timing and duration of intervention	Equity-informed design	Equity-informed target population	Equity-informed outcome evaluation	Narrative description of intervention results
	participatory learning and action group.								but not the food transfer, suggesting that intergenerational bargaining may affect interventions aiming to empower younger women.
**Hazra (2020)**	Implementation of health behaviour change interventions including women's self-help groups for maternal and child health information dissemination, home visits, and community meetings to initiate complementary feeding and training for community health volunteer.	Quasi experimental study	Married women of reproductive age who recently gave birth	8,865 (overall) 4,615 (baseline) 4,250 (endline)	2015 to 2017 (24 months)	Yes	No - all women	Yes - wealth status	The intervention had mixed success, with a significant increase in ≥4 antenatal care visits and postnatal care visits, but no effect on childbirth at a health facility. However, there was no significant effect on any of the 3 outcomes for women from the poorest groups.
**Hewitt (2017)**	Introduction of national universal paid parental leave scheme (18 weeks at minimum wage) for women who were employed before childbirth.	Pre/post study	Women who gave birth and were employed before birth	5,615 (overall) 2,347 (baseline) 3,268 (endline)	November 2010 to December 2012 (25 months)	Yes	Yes - all women working in public and private sectors, full-time and part-time, professional and non-professional	Yes - employment	The intervention had mixed success, with significant increases in both mental and physical component scores after policy implementation. However, only women on permanent and casual contracts (not fixed-term or self-employed) had significantly increased mental component scores, and only women on permanent and fixed-term contracts (not casual or self-employed) had significantly increased physical component scores.
**Jahn (2022)**	Exploring the impact of justice reform (introduction of a state policy limiting prison-time for low-level violations and scaling back harsh mandatory minimum sentences), on use of antenatal care.	Interrupted time series	All births in Pennsylvania	999,503 (overall)	2009 to 2015 (72 months)	Yes	Yes - Black and Hispanic population	Yes - race and ethnicity	The intervention had mixed success: in counties where prison admissions decreased most after the justice reform, there was a significant increase in early prenatal care and significant decrease in inadequate prenatal care. However, in counties where prison admissions increased after the justice reform, there were smaller but significant increases in early prenatal care and no effect on inadequate prenatal care.
**Karim (2015)**	Introduction of two interventions to improve access to community-based maternal and newborn health services. First, the introduction of a health	Cross-sectional study	All women with children ≤1	7,799 (overall) 3,932 (baseline) 3,867 (endline)	December 2008 to December 2010 (24 months)	Yes	No - all women	Yes - wealth status, geographical access, age	The intervention was successful in significantly increasing any antenatal care visits, receiving iron supplementation and tetanus-toxoid ≥2 doses, any birth preparedness measures, childbirth in

Authors (year)	Description of intervention	Study design	Study population	Sample size	Timing and duration of intervention	Equity-informed design	Equity-informed target population	Equity-informed outcome evaluation	Narrative description of intervention results
	extension program by establishing health posts with health extension workers (2 per 5,000 people), providing free primary healthcare, delivering health education, training on health behaviours, and organising community health promoters. Second, the introduction of the last ten kilometres program including training for health extension workers, health messaging to target populations, and review meetings and supervision visits to health extension workers.								health facility, skilled attendance at birth, and any postnatal care.
**Keya (2018)**	Introduction of a maternal health voucher scheme for increasing access to childbirth in health facilities and skilled attendance at home births for women experiencing poverty.	Quasi-experimental study	Women who gave recently gave birth	6,634 (overall)<br>3,300 (baseline)<br>3,334 (endline)	2010 to 2012 (24 months)	Yes	Yes - poor women living in rural areas	Yes - wealth status	The intervention had mixed success, with a significant increase in childbirth in public health facilities, but no effect on increasing overall childbirth in health facilities. Childbirth in health facilities increased in both intervention and control groups, and rich-to-poor equity gaps reduced in both intervention and control groups.
**Kusuma (2016)**	Introduction of two social assistance programs: cash transfers to poor households (PKH) ($60-220/year/ household and additional $80/year for pregnant women and women with children ≤6 years, additional $40/year for women with 1 child in primary school, additional $80/year for women with one child in secondary school), and cash transfers to villages in rural areas (Generasi) ($8,500 in 2007 and $18,200 in 2009 per village) to support health and education.	Randomised trial (cluster randomised trial)	All married women with pregnancies or births in last 2 years and all health providers	16,595 (overall)<br>9,753 (baseline)<br>6,842 (endline)	2007 to 2009 (24 months)	Yes	Yes - poor households and villages in rural areas	Yes - wealth status, age, education	The intervention had mixed success, with significant increases from the PKH intervention on ≥4 prenatal visits, childbirth in health facilities, and 2 postnatal care visits. There was no effect from the PKH intervention on iron supplementation during pregnancy, or from the Generasi intervention on ≥4 prenatal visits or 2 postnatal care visits.

Authors (year)	Description of intervention	Study design	Study population	Sample size	Timing and duration of intervention	Equity-informed design	Equity-informed target population	Equity-informed outcome evaluation	Narrative description of intervention results
**Kuwawenaruwa (2019)**	Implementation of national free health insurance for poor pregnant women.	Evaluation (mixed-methods)	Women who gave birth in last 12 months	6,000 (overall) 3,000 (baseline) 3,000 (endline)	April 2012 to February 2014 (22 months)	Yes	Yes - poor women	No	The intervention was not successful, with no effect on any antenatal care visits, ≥4 antenatal care visits, timing of antenatal care visits, childbirth in health facilities, or timing of postnatal care visits.
**Lewkowitz (2020)**	Introduction of a mobile application containing on-demand video education about breastfeeding.	Randomised trial	Low income, first-time mothers	169 (overall) 84 (intervention) 85 (control)	July 2017 to December 2018 (17 months)	Yes	Yes - poor women	No	The intervention was not successful, with no effect on exclusive breastfeeding.
**Maldonado (2020)**	Introduction of group health education for women facilitated by community health volunteers and microfinance sessions.	Cohort study (prospective, matched-cohort study)	Pregnant women attending first ANC visits at MOH sponsored health facility	326 (overall) 211 (intervention) 115 (control)	October 2012 to December 2013 (14 months)	Yes	No - all women	Yes - age, education, employment	The intervention was successful, with significant increases to childbirth in health facilities.
**Malik (2016)**	Evaluation of the benefits of publicly financed services and spending on maternal, newborn, and child health.	Cross-sectional study	Women who used public health facilities in their last pregnancies	Not specified	2007 to 2011 (48 months)	Yes	No - all women	Yes - wealth status	The intervention had mixed success, with a significant increase in prenatal consultations and reversal of inequity from pro-rich to pro-poor. However, there was no impact on childbirth in health facilities or postnatal consultations.
**Målqvist (2015)**	Facilitation of monthly local stakeholder groups to stimulate problem-solving in relation to maternal and newborn care (PDSA cycles).	Randomised trial (cluster randomised)	Women who gave birth	1,613 (overall) 841 (intervention) 772 (control)	July 2008 to June 2011 (35 months)	Yes	No - all women	Yes - wealth status, race/ ethnicity, education	The intervention had mixed success, with significant reductions in neonatal mortality among poor women, but no effect on neonatal mortality for women with low education or non-poor women.
**McDougal (2017)**	Introduction of the Ananya program, consisting of community outreach services for frontline workers. Frontline workers equipped with mobile kunji (voice response-based mobile services to promote 10 life saving RNMCH behaviours) and mobile academy (mobile training course for frontline workers).	Quasi-experimental study (two-armed)	Married women who gave birth in the last 6 months but are not currently pregnant	13,334 (overall) 3,229 (intervention) 10,105 (control)	Project implementation from 2011, data collection January 2012-April 2014	Yes	No - all women	Yes - wealth status, gender equity	The intervention had mixed success, with no effect on overall rates of ≥4 antenatal care visits, skilled birth attendance, or postnatal care. However, multivariate analysis showed a significant effect of the intervention on continuity of care for the poorest women, women with no or primary education, women's husbands with no or primary education, women from Scheduled Castes or Scheduled Tribes, Muslim women, women married at age <18 years, and women who were excluded from ≥1 household decision.

Authors (year)	Description of intervention	Study design	Study population	Sample size	Timing and duration of intervention	Equity-informed design	Equity-informed target population	Equity-informed outcome evaluation	Narrative description of intervention results
**Mohan (2021)**	Introduction of health information for pregnant women through 1 year post-partum through Kilkari, a maternal mobile messaging program.	Randomised trial	Women who are currently pregnant or postpartum women (up to 1 year) with access to mobile phone	4,423 (overall)<br>2,349 (intervention)<br>2,074 (control)	2017 to 2020 (36 months)	Yes	No - women who have access to mobile phone	Yes - wealth status, caste, education	The intervention had mixed success based on the equity analysis. Disparities in household phone ownership and women's access to phones created inequities in eligible population (marginalised caste groups, less educated women). Health behaviours had different equity distributions, such as sterilisation concentrated among the poor and contraceptive use concentrated among the rich. The intervention closed equity gaps in wealth more than in education, and enabled the 'worse off' to make proportional gains with the 'better off' (absolute gains are preferable due to pre-existing disadvantage).
**Mwase (2022)**	Introduction of 4 types of performance-based financing where health facilities were rewarded by the Ministry of Health for achievement of defined health service indicators using a case-based payment system, adjusted for quality of care after<br>Verification Three out of 4 schemes specifically targeted the ultra-poor.	Randomised trial (cluster randomised trial nested within pre/post study with independent controls)	Women of reproductive age	34,157 (overall)<br>16,291 (baseline)<br>17,866 (endline)	November 2013 to June 2017 (43 months)	Yes	Yes - poor women	Yes - wealth status, age, literacy, geographical access	The intervention had mixed success, with a significant increase in childbirth in health facilities and ≥3 postnatal care visits, but no effect on first antenatal care visit, ≥4 antenatal care visits, tetanus-toxoid ≥2 doses, HIV testing in pregnancy, iron supplementation in pregnancy, or ≥1 postnatal care visits. The positive intervention effect was primarily driven by increases among the richest women.
**Paredes (2016)**	Introduction of two pro-poor policies: universal health care insurance and conditional cash transfers for women with ≥4 antenatal care visits and give birth in a health facility.	Cross-sectional study	Women who gave birth in last <12 months	Not specified	2008 to 2013 (60 months)	Yes	Yes - conditional cash transfer targets poor women	Yes - wealth status, urban/ rural, maternal education	The intervention had mixed success, with significant increases to all women giving birth in health facilities, but no effect on ≥4 antenatal care visits or caesarean section. However, concentration indices show that ≥4 antenatal care visits, childbirth in health facilities, and caesarean section are all remain pro-rich.
**Parmar (2019)**	Introduction of interventions to improve supply-side of maternal health care: emergency obstetric training for nurses, general practitioners, anaesthetists, and	Cross-sectional study	Children born during 1992-2010, DHS survey 1997, 2004, 2010	30,364 (overall)	1997 (12 months)<br>2004 (12 months)	Yes	No - all women	Yes - wealth status, education, urban/rural	The intervention had mixed success, with overall significant increases in childbirth in health facilities and assisted childbirth. However, there were significantly worsening inequalities between poor and

Authors (year)	Description of intervention	Study design	Study population	Sample size	Timing and duration of intervention	Equity-informed design	Equity-informed target population	Equity-informed outcome evaluation	Narrative description of intervention results
	surgical assistants, delegation of emergency obstetric care services to these providers, and free childbirth and caesarean section in public health facilities.				2010 (12 months)				richer populations, suggesting that only better-off women are benefiting from the interventions.
**Paul (2019)**	Evaluation of Janani Suraksha Yojana (JSY), a national roll out of financial support to pregnant women to encourage childbirth at health facilities. There are two types of JSY: Universal JSY targeting all women in low-performing states, and Targeted JSY targeting women living below the poverty line in high-performing states.	Pre/post study	All ever-married women aged 15-49 years.	223,571 (overall)	Project implementation from 2005, data collection in 2005-2006 and 2015-2016	Yes	Yes - poor women	Yes - wealth status, education, castes/tribes, urban/rural	The intervention had mixed effects. Low-performing states with the universal JSY experienced significant declines in inequalities around childbirth in health facilities and postnatal care visits. However, high-performing states with targeted JSY experienced significant declines in inequalities around ≥4 antenatal care visits and comprehensive antenatal care check-ups.
**Powell-Jackson (2009)**	Introduction of conditional cash transfers to all women giving birth in public health facilities and an incentive to the health provider for each birth attended, either at home or in the facility.	Interrupted time series	All pregnant women	14,799 (overall)<br>7,613 (baseline)<br>7,186 (endline)	2001 to 2007 (72 months)	Yes	No - all women	Yes - wealth status, religion, age, caste, education	The intervention had mixed effects, with significant increases in women accessing maternal health services (childbirth at government health facilities, skilled attendance at birth, childbirth with any health worker). However, concentration indices show that richer families have smaller proportions of total household resources on out-of-pocket health expenses, compared to poorer families, suggesting that inequalities persist in household expenditure on maternal healthcare.
**Priedeman Skiles (2013)**	Introduction of performance-based financing for health workers and health facilities to improve equity in maternal health service provision, with payment based on service outputs weighted per facility quality score.	Quasi-experimental study (matched-pair)	Women who gave birth in the last 18 months (ANC, birth) and all women 15-49 years (contraception)	2,044 (overall)	Project implementation from April 2008, data collection 2005 and 2007 DHS	Yes	No - all women	Yes - wealth status, urban/ rural	The intervention had mixed effects, with significant increases in childbirth in health facilities, but no effect on first antenatal care visit or ≥4 antenatal care visits. There was no effect on reducing health inequities.
**Quayyum (2013)**	Introduction of training for community health workers to improve community-based pregnancy registration, support	Quasi-experimental study	Women who were pregnant in the last 12 months	4,190 (overall)<br>2,468 (intervention)<br>1,722 (control)	September 2008 to January 2010 (16 months)	Yes	Yes - women living in rural areas	Yes - wealth status	The intervention had mixed effects, with overall significant increases to ≥4 antenatal care visits, childbirth in health facilities, childbirth at home with skilled birth

Authors (year)	Description of intervention	Study design	Study population	Sample size	Timing and duration of intervention	Equity-informed design	Equity-informed target population	Equity-informed outcome evaluation	Narrative description of intervention results
	for maternal healthcare services, and strengthen referrals.								attendant, and first postnatal care visit. Equity analyses showed that rich-to-poor ratios significantly reduced after the intervention, but remained pro-rich for all indicators.
**Randive (2014)**	Evaluation of Janani Suraksha Yojana (JSY), a cash incentive program for pregnant women to give birth in public health facilities, with women in rural areas receiving USD$31 and women in urban areas receive USD$22.	Cross-sectional study	Women who were pregnant and gave birth	Not specified	Project implementation from 2005, data collection in 2004-2006 and 2010	Yes	Yes - all women	Yes - wealth status	The intervention had mixed effects, with reduced but persisting inequalities in childbirth in health facilities, and decreased in maternal mortality significantly slower in the poorest areas compared to the richest areas.
**Reeve (2016)**	Introduction of midwifery-led interdisciplinary outreach services, including employment of Aboriginal health workers, fortnightly visits to the community by a midwife completing her midwifery training, and monthly visit by general practice obstetricians.	Cohort study (retrospective using medical records)	All Indigenous women who were pregnant or gave birth	213 (overall)<br>92 (baseline)<br>121 (endline)	April 2007 to September 2011 (53 months)	Yes	Yes - Indigenous women	No	The intervention had mixed effects, with significant increases in first antenatal care visits and some improvements to screening for alcohol intake, smoking, and ultrasound scan), but no effect on mode of birth, low birthweight or preterm birth.
**Sandner (2018)**	Introduction of an intensive targeted home visiting program (Pro Kind) for maternal and child health.	Randomised trial	First time mothers experiencing financial and social disadvantage	755 (overall)<br>394 (intervention)<br>361 (control)	2006 to 2012 (72 months)	Yes	Yes - women experiencing financial and social	No	The intervention had mixed effects, with small improvements to maternal mental health, but no effect of home visiting on health utilisation, health behaviours, or maternal physical health.
**Sarmiento (2022)**	Introduction of interventions to support the provision of cultural safety during childbirth by providing stipends and training for traditional midwives, training for staff in local government health centres and training for young community members to support traditional midwives.	Randomised trial (pragmatic parallel-group cluster-randomised controlled non-inferiority trial)	All women from four Indigenous groups who gave birth or were pregnant, and their family members	872 (overall)<br>404 (intervention)<br>468 (control)	February 2015 to May 2017 (27 months)	Yes	Yes - Indigenous women	No	The intervention had mixed effects, with significant increases in traditional childbirth (at home, with traditional midwife and family present and in preferred vertical position). However, there was no effect on perinatal death, neonatal complications, or caesarean section.
**Schuler (2015)**	Introduction of interactive workshops with couples to	Randomised trial	Women and men who are married or in	1,122 (overall)<br>1,122 (baseline)<br>603 (endline)	Project implementation: 2011-2012, data	Yes	Yes - Indigenous women	Yes - gender equity scores	The intervention had mixed effects, with significant improvements in gender

Authors (year)	Description of intervention	Study design	Study population	Sample size	Timing and duration of intervention	Equity-informed design	Equity-informed target population	Equity-informed outcome evaluation	Narrative description of intervention results
	promote gender equity and contraception.		civil unions and live in the community		collection: March-April 2012				attitudes, but no effect on contraceptive use among couples.
**Spangler (2014)**	Introduction of a community-based educational intervention on maternal and newborn health provided by community-based health workers (MaNHEP) in rural Ethiopia.	Cross-sectional study	Women who gave birth in the last year	2,046 (overall) 1,027 (baseline) 1,019 (endline)	June 2010 to July 2012 (25 months)	Yes	Yes - women living in rural areas	Yes - wealth status, education, age	The intervention had mixed effects, with exposure to the MaNHEP programme significant associated with far distance to health facilities, previous pregnancy loss, region, and number of antenatal care visits. However, there was no association of exposure to the MaNHEP programme with age, parity, or education.
**Vellakkal (2017)**	The National Rural Health Mission is a programme to support low-income pregnant women for childbirth in health facilities, by increasing public health funding, decentralising village and district level health planning and management, strengthening the public health service delivery infrastructure, and promoting the non-profit sector to increase social participation and community empowerment.	Quasi-experimental study (quasi-natural experiment)	Married women who recently had live birth, stillbirth, or spontaneous or induced abortion	732,358 (overall)	Survey: January 2012 to April 2014 (27 months) community-based household survey data: 2012 to 2017 (60 months)	Yes	Yes - poor women living in the less-developed high-focus Indian states	Yes - wealth status, place of residence (urban/rural), education, castes	The intervention had mixed effects, with inequities in childbirth in health facilities declining after the introduction of the intervention across all socioeconomic groups, and the greatest effect among the lowest and middle wealth and education tertiles. However, there was no effect on antenatal care uptake.
**Ward (2020)**	Introduction of the Ananya programme promoting innovations in interventions and delivery platforms to improve quality, uptake, and equity of key health behaviours.	Pre/post study	Women who gave birth in the last 12 months	68,134 (overall - survey & community-based survey); 10,174 (baseline survey); 9,611 (endline survey) 48,349 (community-based survey)	Survey: January 2012 to April 2014 (27 months) Community-based household survey data: 2012 to 2017 (60 months)	Yes	No - all women	Yes -wealth status, caste	The intervention had mixed effects, with the least marginalised women performing better on desired health behaviours compared to most marginalised women, and inequities persisting over time. Inequities between most and least marginalised women significantly increased for identification of skilled birth attendant, initiation of skin-to-skin newborn care, and significantly decreased for immediate breastfeeding.
**Warren (2015)**	Introduction of a voucher scheme for low-income women to access antenatal care, childbirth, and postnatal care.	Quasi experimental study	Women who recently gave birth	688 (overall) 451 (intervention) 237 (control)	2010 (not specified)	Yes	Yes - poor women	No	The intervention was not successful, with no effect on any measures associated with postnatal care (timing, time spent with provider, and satisfaction).

Authors (year)	Description of intervention	Study design	Study population	Sample size	Timing and duration of intervention	Equity-informed design	Equity-informed target population	Equity-informed outcome evaluation	Narrative description of intervention results
**Willey (2022)**	Introduction of government-led maternal and newborn health interventions including: quality improvement teams in primary health centres, village health worker links with facilities, forums with mothers-in-law and men, mothers groups, task shifting and training community health extension workers and village health workers, financial incentives for village health workers, and improving supply chains.	Quasi-experimental study (plausibility study)	Women who gave birth in last 12 months	3,805 (overall)<br>1,011 (baseline)<br>2,794 (endline)	2016 to 2019 (36 months)	Yes	Yes - all women, but targeting a rural, poor state with high rates of maternal and newborn mortality	Yes - wealth status	The intervention had mixed effects, with improvements to ≥1 and ≥4 antenatal care visits, basic antenatal care quality, childbirth in health facilities, and early postnatal care, however, there were also significant improvements to all outcomes in the control group. Equity analyses show wide equity gaps for all outcomes, favouring the rich.
**Witter (2016)**	Introduction of policies to reduce financial barriers to obstetric care in West Africa, including free caesarean sections (Benin, Mali), 80% subsidies for caesarean section and birth complications (Burkina Faso), and free childbirth, caesarean section, and transportation (Morocco).	Evaluation (comparative case study with realist evaluation)	Women who gave birth	13,3841 (overall)<br>36,375 (Benin)<br>36,836 (Burkina Faso)<br>43,952 (Mali)<br>16,678 (Morocco)	Benin: 1993-2011 (18 years), Burkina Faso: 1988-2010 (22 years), Mali: 1993-2013 (20 years), Morocco: 1998-2011 (13 years)	Yes	No - all women	No	The interventions had mixed success, with the policies effective in meeting financial protection goals and possibly health and equity goals at sustainable cost. However, there was no effect on caesarean section rates or childbirth in health facilities.
**Yelland (2020)**	Introduction of the Bridging the Gap partnership to improve maternity care for families from refugee backgrounds, by adding refugee status to medical records, professional development (clinicians, managers, front-of-house staff), group antenatal care, and professional interpreters.	Interrupted time series	Women of refugee backgrounds who gave birth, and Australian-born women giving birth at same sites at same time	42,142 (overall)<br>4,154 (intervention)<br>37,988 (control)	2014 to 2016 (24 months)	Yes	Yes - refugee women	Yes - refugee status, age	The intervention had mixed effects, with similar trends of improvement in ≥7 antenatal care visits for both women from refugee backgrounds and Australian-born women, and similar trends in decreasing first antenatal care visits at <16 weeks gestation.
